# Stabilization of Two Radicals with One Metal: A Stepwise Coupling Model for Copper-Catalyzed Radical–Radical Cross-Coupling

**DOI:** 10.1038/srep43579

**Published:** 2017-03-08

**Authors:** Xiaotian Qi, Lei Zhu, Ruopeng Bai, Yu Lan

**Affiliations:** 1 School of Chemistry and Chemical Engineering, Chongqing University, Chongqing 400030, China

## Abstract

Transition metal-catalyzed radical–radical cross-coupling reactions provide innovative methods for C–C and C–heteroatom bond construction. A theoretical study was performed to reveal the mechanism and selectivity of the copper-catalyzed C–N radical–radical cross-coupling reaction. The concerted coupling pathway, in which a C–N bond is formed through the direct nucleophilic addition of a carbon radical to the nitrogen atom of the Cu(II)–N species, is demonstrated to be kinetically unfavorable. The stepwise coupling pathway, which involves the combination of a carbon radical with a Cu(II)–N species before C–N bond formation, is shown to be probable. Both the Mulliken atomic spin density distribution and frontier molecular orbital analysis on the Cu(II)–N intermediate show that the Cu site is more reactive than that of N; thus, the carbon radical preferentially react with the metal center. The chemoselectivity of the cross-coupling is also explained by the differences in electron compatibility of the carbon radical, the nitrogen radical and the Cu(II)–N intermediate. The higher activation free energy for N–N radical–radical homo-coupling is attributed to the mismatch of Cu(II)–N species with the nitrogen radical because the electrophilicity for both is strong.

Free radicals, which have an unpaired electron, are unique yet important species in organic reactions^1^,^2^,^3^,^4^. The single electron in one orbital makes them unstable and very reactive^5^,^6^. As high reactivity is often associated to low selectivity, one of the problematic topics for radical chemistry is the side reactions in transformations involving radicals, such as the homo-coupling of free radicals in radical–radical coupling reactions^7^,^8^,^9^. The stabilization of free radicals is necessary to avoid these problems. Fortunately, the introduction of a transition metal into the radical combination reactions provides an efficient approach to stabilize the radicals and protect them from being quenched by homo-coupling or fragmentation^10^,^11^,^12^,^13^,^14^. Moreover, the interplay between a transition metal and radicals would be able to tune the reactivity of radicals and enable specific radical–radical cross-combination^10^,^15^,^16^,^17^. Transition metal-mediated or -catalyzed radical–radical cross-coupling is thereby established as an innovative strategy for the construction of carbon–carbon and carbon–heteroatom bonds^10^,^18^,^19^,^20^,^21^,^22^.

The radical–radical cross-coupling reactions have recently attracted much attention. MacMillan reported that a synergistic combination of photoredox catalysis and organocatalysis could afford radical–radical cross-coupling^23^,^24^,^25^,^26^. Meanwhile, a cross-combination of carbon radicals was also realized by Tunge^27^ and Xiao^28^ through the merger of photoredox catalysis and palladium catalysis. In addition, Lei and co-workers developed a series of transition metal-catalyzed radical–radical cross-coupling reactions (Fig. 1)^16^,^20^,^29^,^30^. Selective carbon–nitrogen, carbon–carbon, and carbon–phosphorus radical–radical cross-coupling would occur with the assistance of transition metals. Undoubtedly, these reactions provide new protocols for organic synthesis; whereas the mechanism behind them is more appealing and challenging. The questions remain as to why this type of reaction might proceed in a selective cross-coupling manner and how might the homo-coupling of radicals be avoided.

Almost all the mechanisms of radical–radical cross-coupling reactions are explained by the persistent radical effect, which requires that two different types of radicals are generated at similar rates and one is more persistent than the other^31^,^32^,^33^,^34^. For transition metal-catalyzed radical–radical cross-coupling, however, the stability and reactivity of radicals would be changed in the presence of metals, which probably does not meet the criteria of the persistent radical effect^16^,^35^. On another hand, the direct combination of two radicals might pass through a spin-crossover process with energy barrier when the spin state of the reactants is a triplet and the coupling product is a singlet (Fig. 2). In this case, the energy barrier could be represented by the minimum energy crossing point (MECP)^36^,^37^,^38^, the energy of which plays a significant role in determining the reaction mechanism and selectivity^38^,^39^,^40^,^41^,^42^. Surprisingly, these issues have seldom been discussed in transition metal-catalyzed radical–radical cross-coupling reactions. More effort should be devoted to reveal the detailed reaction pathway and the origin of the cross-combination selectivity.

Here, we describe our theoretical understanding of transition metal-catalyzed radical–radical cross-coupling reactions^43^,^44^,^45^. As shown in Fig. 3, for a catalytic system that contains two different radical species (R**·** and R’**·**), a low oxidation state transition metal would preferentially coordinate with one radical (for example, the radical R**·**) and combination with the other radical would be less favored. The generated organometallic intermediate M^n+1^−R could be regarded as a metal-containing radical species. Subsequent reaction between this radical and R’**·** has two competitive paths. In the concerted coupling pathway, radical R’**·** would directly attack the R moiety of M^n+1^–R, and the formation of an R–R’ bond and homolytic cleavage of a M–R bond occurs synchronously through the MECP. While in the stepwise coupling pathway, radical R’**·** would initially combine with the metal center and form a high oxidation state organometallic complex through the MECP; that is the transition metal simultaneously stabilizes two radical species. The product R–R’ is finally obtained by a two electron transfer process. Although the concerted radical–radical coupling mode is usually proposed in previous work^20^,^46^, our computational results reveal that the stepwise coupling mechanism is more favored for transition metal-catalyzed radical-radical cross-coupling. Here, the copper-catalyzed oxidative C(sp3)–H/N–H coupling of sulfoximine with cyclohexane (Fig. 4) is studied using density functional theory (DFT) calculations as an example to prove this new viewpoint^21^. Both the mechanism and selectivity of this reaction will be discussed in this work.

## Computational Methods

All the DFT calculations were carried out with the GAUSSIAN 09 series of programs^47^. DFT method B3-LYP^48^,^49^ with a standard 6–31G(d) basis set (SDD^50^ basis set for Cu) was used for geometry optimizations. Harmonic vibrational frequency calculations were performed for all of the stationary points to confirm if the points were local minima or a transition structure and to derive the thermochemical corrections for the enthalpies and free energies. In this study, the stability of wavefunction has been tested for singlet, doublet, and triplet state intermediates. All the test results confirmed that the wavefunction is stable under the perturbations considered. The solvent effects were considered by single point calculations on the gas-phase stationary points with an SMD continuum solvation model^51^. The M06 functional^52^ with the 6–311+G(d, p) basis set (def2-TZVP basis set for Cu)^42^ was employed to calculate the solvation single point energies in a cyclohexane solvent to provide more accurate energy information^53^,^54^,^55^,^56^. The Gibbs free energy of each stationary point calculated by M06 is provided for discussion of the energy. The Mulliken atomic spin density of certain atoms was also calculated using the same method. The optimized structures were displayed using CYLview^57^.

Additionally, the MECP location program^58^,^59^,^60^ developed by Harvey and co-workers was used in this study to gain the structures of MECPs at the B3-LYP/6–31G(d) (SDD basis set for Cu) level of theory. The single point energies of the MECPs, which were calculated at M06/6–311+G(d,p) (def2-TZVP basis set for Cu) in cyclohexane, have also been determined. The global electrophilicity *ω*° and global nucleophilicity *N*°, which are employed to measure the electron compatibility of free radicals, were gained at the UB3-LYP/6–31G(d) (SDD for Cu) level using^61^,^62^,^63^:


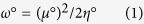



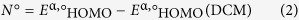


where *μ*° is the global chemical potential of radicals, which could be obtained by *μ*° ≈ (*E*^α,°_HOMO_^ + *E*^β,°_LUMO_^)/2, and *η*° is the global chemical hardness of radicals, which could be obtained using η° ≈ (*E*^β,°_LUMO_^ − *E*^α,°_HOMO_^). The *E*^α,°_HOMO_^ and *E*^β,°_LUMO_^ are corresponding orbital energies of the HOMO in α molecular orbitals and the LUMO in β molecular orbitals. In Eq. 2, DCM represents a dicyanomethyl radical^61^,^62^,^63^.

## Results and Discussion

**Figure 5:**
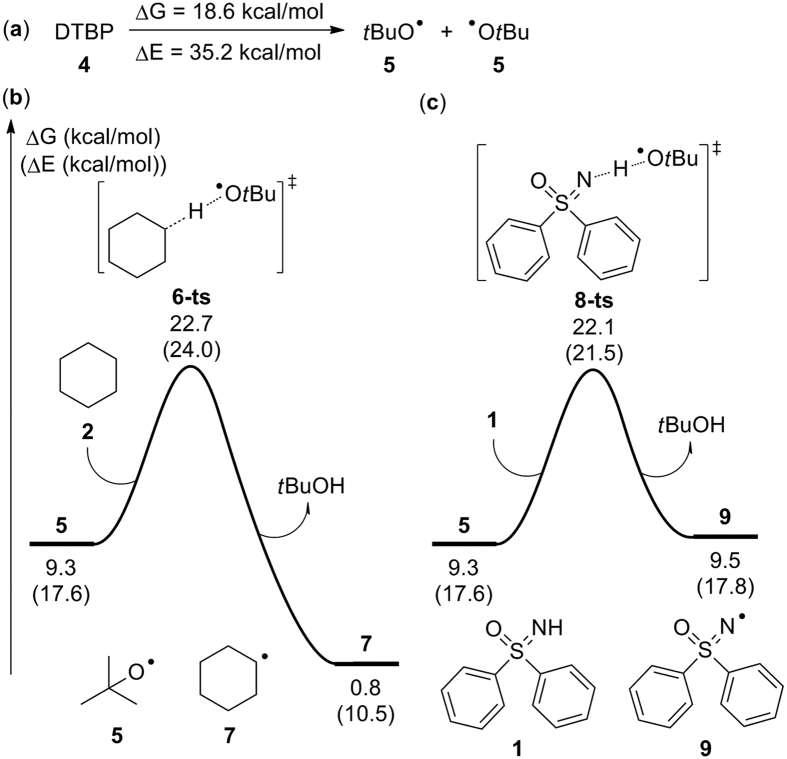
Calculated free energies for (**a**) the homolysis of di-*tert*-butyl peroxide (DTBP), (**b**) the formation of carbon radical **7**, and (**c**) the formation of nitrogen radical **9**.

In the selected copper-catalyzed C–N coupling reaction, di-*tert*-butyl peroxide (DTBP), which could decompose to *tert*-butoxyl radicals, was used as the radical initiator^21^. Reactants sulfoximine **1** and cyclohexane **2** could react with the *tert*-butoxyl radical, leading to the generation of a nitrogen radical and a carbon radical, respectively. The C–N bond is considered to be constructed through radical–radical cross-coupling. In our theoretical calculation, the formation of a carbon radical and a nitrogen radical was first studied. As the homolysis of DTBP (**4**) is endergonic by 18.6 kcal/mol, the relative free energy of each *tert*-butoxyl radical (**5**) was determined to be 9.3 kcal/mol (Fig. 5a). Subsequent radical substitution between radical **5** and cyclohexane **2** resulted in the generation of carbon radical **7** via transition state **6-ts** (Fig. 5b); the activation free energy for the whole process was 22.7 kcal/mol. The free energy of carbon radical **7** was found to be 0.8 kcal/mol relative to DTBP. An analogous process between radical **5** and reactant **1** could generate nitrogen radical **9** through **8-ts** with an activation free energy of 22.1 kcal/mol (Fig. 5c), which is only 0.6 kcal/mol lower than that of the formation of carbon radical **7**. The formation of radical **9** was determined to be endergonic by 9.5 kcal/mol. Therefore, both the carbon radical and the nitrogen radical are able to be generated in this reaction and carbon radical **7** is more stable than nitrogen radical **9**.

**Figure 6:**
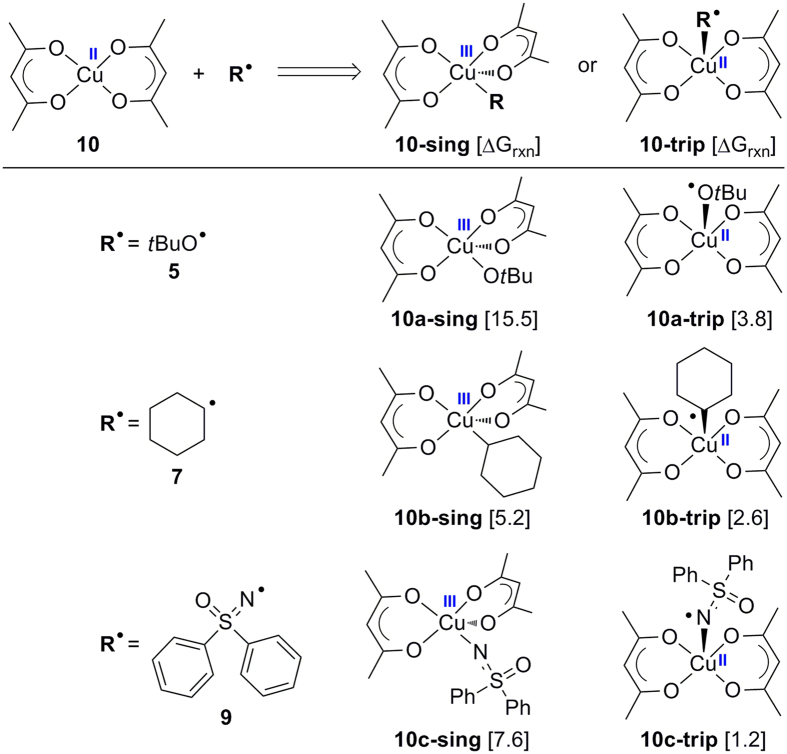
Interaction between Cu(acac)_2_ and different radical species.

In the experiment, the addition of Cu(acac)_2_ facilitated the transformation^21^. To determine its role in this reaction, the interaction between the existing radicals and Cu(acac)_2_ was studied. A radical could either oxidize the metal by forming a covalent bond or purely coordinate with the metal; both of these two cases have been considered in Fig. 6. Complex **10a-sing**, **10b-sing**, and **10c-sing** are determined to be Cu(III) complex because the Wiberg bond index (WBI) bond orders of newly formed Cu–O, Cu–C, Cu–N are 0.67, 0.62, 0.73, respectively. These WBI values imply that new covalent bonds are formed when these singlet intermediates are generated. Moreover, the sum of individual WBI of copper in **10a-sing**, **10b-sing**, and **10c-sing** is found to be 2.39, 2.15, and 2.43, respectively. Thus, these three complexes all involve a Cu(III) center. From the energy point of view, the combination of a *tert*-butoxyl radical with Cu(acac)_2_, which forms a singlet Cu(III) complex **10a-sing**, was endergonic by 15.5 kcal/mol. Meanwhile, the generation of a triplet Cu(II) complex **10a-trip** through radical coordination was endergonic by 3.8 kcal/mol, which is 11.7 kcal/mol lower than the formation of **10a-sing**. For the interplay between carbon radical **7** and Cu(acac)_2_, the formation of Cu(III) complex **10b-sing** or Cu(II) complex **10b-trip** were endergonic by 5.2 or 2.6 kcal/mol, respectively. Moreover, the tendency of free energy for the interaction between nitrogen radical **9** and Cu(acac)_2_ remained the same. These data indicate that Cu(acac)_2_ would rather coordinate with these radical species than form a Cu(III) complex under these conditions. The generation of *tert*-butoxide anion and a Cu(III) complex from **10a-sing** is also determined to be endothermic by 77.9 kcal/mol (See SI for details). Consequently, the mechanism proposed in the experiment that involves the formation of *tert*-butoxide anion and a Cu(III) complex could be safely ruled out. Most importantly, the energy information obtained for radical coordination has shown that the catalyst Cu(acac)_2_ does not have the capability of stabilizing radicals **5**, **7**, or **9**.

**Figure 7:**
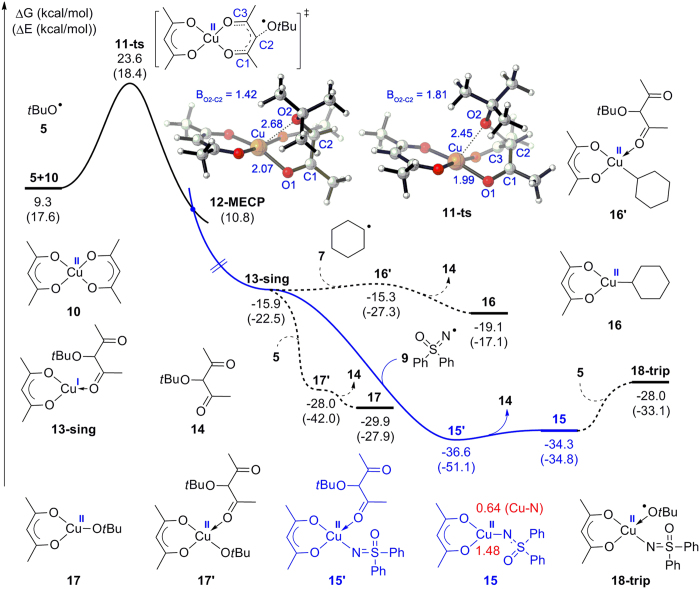
Free energy profile for the formation of active catalytic species 15 through 12-MECP. The red numbers are the Wiberg bond index (WBI) bond orders of Cu–N and the sum of WBI values of Cu in complex **15**.

Both theoretical and experimental studies have suggested that a Cu(I) species might be the active catalyst in copper-catalyzed coupling reactions^64^,^65^,^66^,^67^. Lei and co-workers observed a Cu(I) β-diketonate species by Operando IR and X-ray absorption spectroscopy, which is proven to be the active catalyst for the copper-catalyzed carbon–carbon coupling reaction^68^. Inspired by these studies, we propose that an active β-diketone Cu(I) complex, which could be formed from Cu(acac)_2_, might be the real catalytic species formed in this C–N coupling reaction. As shown in Fig. 7, the nucleophilic addition of *tert*-butoxyl radical **5** towards the middle carbon (C2) of the diketonate through transition state **11-ts** would result in a reduction of Cu(acac)_2_. The generated Cu(I) complex **13-sing** was determined to be singlet because the free energy of the corresponding triplet compound is 29.1 kcal/mol higher than that of **13-sing** (See Figure S3 for details). As the spin state of the reactants before the reduction is a triplet, a minimum energy crossing point **12-MECP** is located between **11-ts** and **13-sing**. The relative energy of **12-MECP** is found to be 10.8 kcal/mol. Optimized structures, shown in Fig. 7, suggest that the length of the O2–C2 bond in **12-MECP** is 1.42 Å, which is 0.39 Å shorter than that in **11-ts** (1.81 Å). Moreover, the distance of Cu–O2 in **12-MECP** is 0.23 Å longer than that in **11-ts**. These data confirm that **12-MECP** occurs later relative to transition state **11-ts**.

Subsequent coordination of nitrogen radical **9** toward Cu(I) complex **13-sing** would generate a Cu(II) intermediate **15′**. The dissociation of complex **14** then forms Cu(II) β-diketonate intermediate **15**, the relative free energy of which is found to be −34.3 kcal/mol. Meanwhile, the WBI value of newly formed Cu–N in **15** is shown to be 0.64 (Fig. 7), suggesting this bond is a covalent bond. Moreover, the sum of WBI values of Cu is 1.48, which further confirms that the oxidation state of copper in complex **15** is +2. Likewise, carbon radical **7** and oxygen radical **5** may combine with the copper center, generating cyclohexyl Cu(II) **16** and *tert*-butoxyl Cu(II) **17** through associated intermediate **16′** and **17′**, respectively. Although the formations of different Cu(II) complexes are all exergonic, the relative free energy of **16** is 10.8 kcal/mol higher than that of **17** while the relative free energy of **17** is 4.4 kcal/mol higher than that of **15**, which indicates that the binding capability of nitrogen radical **9** with Cu(I) is the strongest. In addition, the coordination of the *tert*-butoxyl radical to Cu(II) intermediate **15** was also taken into account. The formation of a triplet Cu(II) complex **18-trip** (the relative free energy of singlet structure was found to be 2.6 kcal/mol higher, see Figure S4 for details) was endergonic by 5.7 kcal/mol, which is energetically unfavorable. Obviously, subsequent transformation would not occur through complex **18-trip**. Therefore, the Cu(I) species **13-sing** would preferentially stabilize the nitrogen radical in this reaction, and the Cu(II)–N β-diketonate intermediate **15** is most likely to be the startup species for the following transformation.

**Figure 8:**
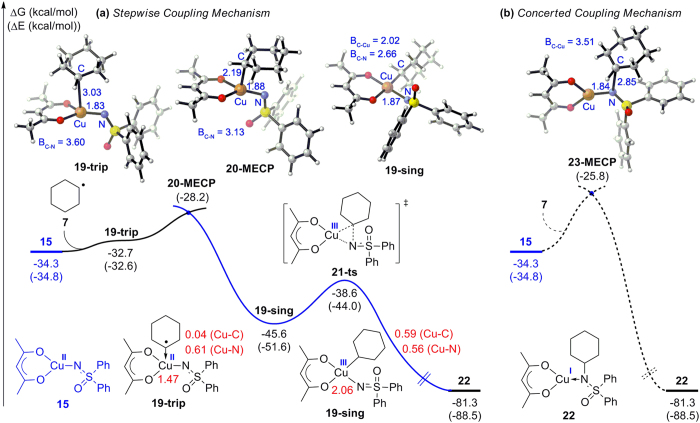
Free energy profiles of the (**a**) stepwise and (**b**) concerted coupling pathway for the C–N radical–radical cross-coupling. The red numbers are the Wiberg bond index (WBI) bond orders of Cu–C, Cu–N and the sum of WBI values of Cu.

For the radical–radical combination of Cu(II) intermediate **15** with carbon radical **7**, two possible pathways were calculated and are summarized in Fig. 8. In the stepwise coupling pathway (Fig. 8a), carbon radical **7** could initially coordinate to Cu(II) intermediate **15**, forming a triplet Cu(II) complex **19-trip** with a free energy increase of 1.6 kcal/mol. The corresponding singlet state, which is more stable by 12.9 kcal/mol, could also be obtained as Cu(III) complex **19-sing** through a minimum energy crossing point **20-MECP**. The relative energy of **20-MECP** is determined to be −28.2 kcal/mol, which is merely 6.6 kcal/mol higher than Cu(II) intermediate **15**. The formation of Cu(III) intermediate **19-sing** is thereby thermodynamically favorable and kinetically feasible. Structural analysis shows that the newly forming C–Cu bond in **19-trip** reaches a length of 3.03 Å, while this bond length decreased to 2.19 and 2.02 Å in **20-MECP** and **19-sing**, which implies that the interaction between carbon radical and the metal center becomes stronger along the reaction coordinate. Besides, the WBI bond order of Cu–C in **19-trip** is determined to be 0.04, which means almost no covalent interaction exist between Cu and C. In complex **19-sing**, the sum of WBI values of Cu is 2.06, confirming that the oxidation state of copper is +3. Moreover, the WBI bond order of Cu–C in **19-sing** is found to be 0.59, which suggests this Cu–C is a strong covalent bond. The structural analysis and bond order results thereby account for the high stability of singlet state **19-sing**. The C–N bond is then constructed through a two-electron transfer/reductive elimination transition state **21-ts**, and Cu(III) is reduced to Cu(I) simultaneously. The activation free energy of this step is determined to be only 7.0 kcal/mol, and the formation of Cu(I) complex **22** is exergonic by 35.7 kcal/mol.

**Figure 9:**
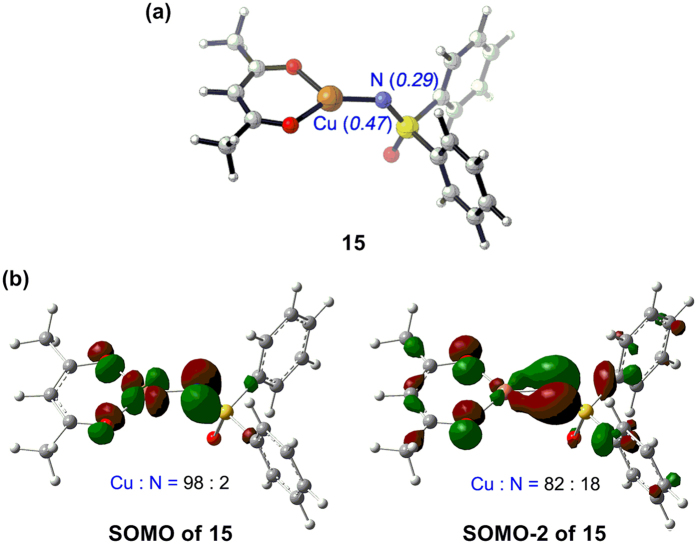
(**a**) Optimized structure of Cu(II) intermediate **15**. The italic numbers in parentheses are the corresponding Mulliken atomic spin density of certain atoms. (**b**) Calculated SOMO and SOMO-2 of **15** at ROB3LYP/6–31 G (**d**) level of theory. The numbers in black are the proportions of molecular orbitals on Cu and N.

In the concerted coupling pathway (Fig. 8b), the C–N bond was formed via the nucleophilic addition of carbon radical **7** toward the nitrogen atom in Cu(II) intermediate **15**. A minimum energy crossing point between the triplet and singlet potential energy surfaces was located as **23-MECP**, the relative energy of which was found to be −25.8 kcal/mol. Comparing these two pathways reveal that the concerted coupling mechanism is kinetically unfavorable, and the carbon radical would prefer to combine with the metal center before C–N bond formation. Further theoretical analysis has been carried out to elucidate the preference of the carbon radical for Cu(II). As shown in Fig. 9a, the Mulliken atomic spin density of copper atom in intermediate **15** is 0.47 while the corresponding value of the nitrogen atom is only 0.29, which indicates that the spin is mainly located at the metal center. Moreover, the singly occupied molecular orbital (SOMO) of **15** is determined to be the antibonding orbital of Cu–N π bond (Fig. 9b), and the corresponding bonding orbital is observed as SOMO-2. Thus, Cu(II) intermediate **15** should be an electron-donating radical species. On another hand, the proportion of SOMO on the Cu atom is calculated to be 0.98, which is much higher than that on N atom. Both the Mulliken atomic spin density distribution and frontier molecular orbital (FMO) analysis have proven that the combination ability of Cu with another radical is stronger than that of N. Consequently, the carbon radical would preferentially react with the metal center rather than nitrogen.

**Figure 10:**
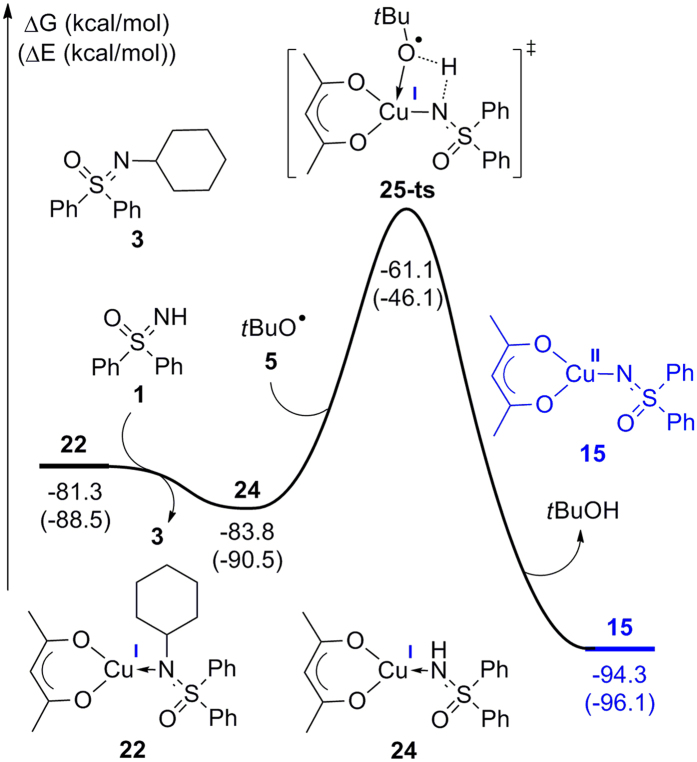
Free energy profile for the regeneration of Cu(II) intermediate 15.

When the Cu(I) complex **22** was formed in this reaction, the C–N coupling product **3** would be finally obtained by ligand exchange using reactant **1** (Fig. 10). Generated Cu(I) species **24** with the coordination of reactant **1** would then react with *tert*-butoxyl radical **5**, thereby regenerating the active Cu(II) species **15** by hydrogen transfer through a four-membered ring transition state **25-ts**. The activation free energy was determined to be 22.7 kcal/mol, which is very close to the metal-free radical substitution transition state **8-ts** (22.1 kcal/mol).

**Figure 11:**
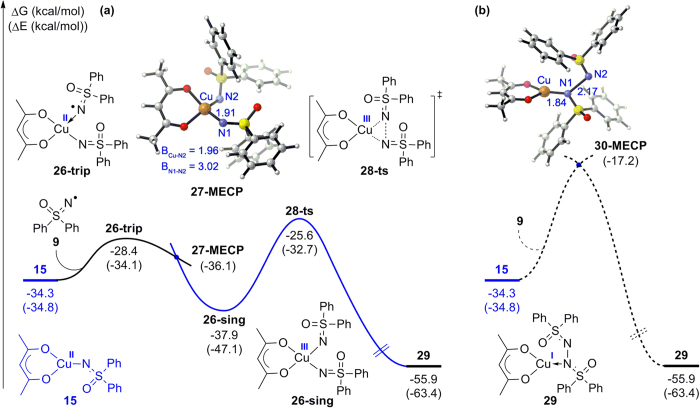
Free energy profiles of the (**a**) stepwise and (**b**) concerted coupling pathway for the N–N radical–radical homo-coupling.

With the reliable mechanism in hand, we wanted to understand why the N–N homo-coupling did not take place in this reaction and sought to determine what would happen if the carbon radical **7** in the cross-coupling pathway was replaced with nitrogen radical **9**. Computational results for these issues are shown in Fig. 11. Along the stepwise homo-coupling pathway (Fig. 11a), a triplet Cu(II) complex **26-trip** would be formed with a free energy increase of 5.9 kcal/mol after the coordination of another nitrogen radical **9** to **15**. The free energy barrier for this step is found to be 4.3 kcal/mol higher than that of the carbon radical coordination shown in Fig. 8a, which implies the coordination capability of a nitrogen radical to Cu(II) is weaker than that of a carbon radical. Subsequently, through a minimum energy crossing point **27-MECP**, the triplet Cu(II) species **26-trip** could be transformed to a singlet Cu(III) complex **26-sing**, the formation of which is exergonic by 3.6 kcal/mol relative to **15**. This process is less favored compared with the generation of **19-sing** (Fig. 8a), which is exergonic by 11.3 kcal/mol. It is also noteworthy that the N–N bond formation via transition state **28-ts** bears an activation free energy of 12.3 kcal/mol, and this value is 5.3 kcal/mol higher than that of the C–N bond formation transition state **21-ts**. Additionally, the energy barrier of the minimum energy crossing point **30-MECP** in the concerted coupling pathway reaches up to 17.6 kcal/mol (Fig. 11b), which is 3.2 kcal/mol higher than the activation energy through **28-ts** (14.4 kcal/mol). These data confirms that the homo-coupling of nitrogen radicals could not take place in this reaction and also verify the preceding conclusion that the radical–radical coupling reactions would not proceed along the concerted coupling pathway.

**Figure 12:**
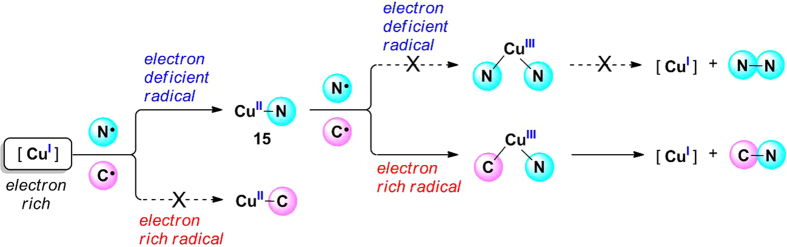
Graphic illustration for the origin of cross-coupling selectivity.

We have demonstrated that the N–N radical–radical homo-coupling is less favored compared with the C–N radical–radical cross-coupling because the bonding capacity of Cu(II) to a nitrogen radical is weaker than that to a carbon radical. An interesting phenomenon then arises from above study, which is that the low oxidation state Cu(I) species tends to combine with a nitrogen radical rather than a carbon radical; however, the generated high oxidation state Cu(II) species prefers to combine with the carbon radical instead of the nitrogen radical. Hence, copper could selectively stabilize two diverse radical species at different oxidation states. The principle behind this finding plays a vital role in determining the selectivity of radical–radical coupling reactions. Two reactivity indices that were introduced by Domingo and co-workers for free radicals, the global electrophilicity *ω*° and global nucleophilicity *N*°^62^, have been used to clarify the origin of the selectivity in this work (Table 1)^69^,^70^,^71^, and a graphic illustration for the cross-coupling selectivity is also provided based on these data (Fig. 12).

As shown in Table 1, the global electrophilicity *ω*° of carbon radical **7** is 0.99 eV while the *ω*° of nitrogen radical **9** is 3.54 eV, which suggests that the nitrogen radical **9** is a strong electrophile and carbon radical **7** is only a moderate electrophile. However, the global nucleophilicity *N*° of carbon radical **7** is calculated to be 3.06 eV, indicating that this radical is a strong nucleophile. Thus, the electron rich Cu(I) complex would preferentially combine with the electron deficient nitrogen radical **9** (Fig. 12). For the generated Cu(II)–N intermediate **15**, which is a metal-contained radical species, the global electrophilicity *ω*° is determined to be 3.12 eV. As a strong electrophile, the combination of **15** with the electron deficient nitrogen radical is obviously unfavorable (Fig. 12). The higher energy barrier for N–N bond formation could also be attributed to the strong electrophilicity of nitrogen radical **9**. In contrast, the combination of electrophile **15** with a nucleophilic carbon radical is energetically favorable. Subsequent C–N bond formation is also a facile process because the nucleophilicity of carbon radical was matched with the electrophilicity of the nitrogen radical. As a consequence, the chemoselectivity of cross-coupling in this study originates from the suitable matching of the electrophilicity of Cu(II)–N intermediate **15** with the strong nucleophilicity of carbon radical **7**, as well as the matching of the nucleophilic carbon radical with the electrophilic nitrogen radical.

## Conclusion

A stepwise coupling mechanism, where a metal center is able to successively stabilize two radical species, has been proposed and proven to be probable for copper-catalyzed C–N radical–radical cross-coupling reaction. The catalytic cycle starts with the coordination of the nitrogen radical to a Cu(I) complex, which is formed by the reduction of Cu(acac)_2_ through a MECP, generating an active Cu(II)–N β-diketonate intermediate. This Cu(II)–N species preferentially combines with a carbon radical and forms a Cu(III) complex through another MECP. A subsequent two-electron transfer/reductive elimination process constructs the C–N bond. The concerted coupling pathway, in which the formation of a C–N bond and cleavage of a Cu(II)–N bond occurs simultaneously by the nucleophilic addition of a carbon radical to the nitrogen in a Cu(II)–N species, has been demonstrated to be unfavorable. Because both the Mulliken atomic spin density distribution and FMO analysis on this active Cu(II)–N species have shown that the Cu site is more reactive than the N site, the carbon radical tends to react with the metal center rather than nitrogen moiety.

Moreover, the calculations of global electrophilicity *ω*° and nucleophilicity *N*° for the carbon radical, the nitrogen radical, and the Cu(II)–N β-diketonate intermediate suggest that the carbon radical is a strong nucleophile while the nitrogen radical and the Cu(II)–N β-diketonate intermediate are strong electrophiles. The matching of the electrophilicity of the Cu(II)–N intermediate with the strong nucleophilicity of the carbon radical makes it easier for the combination of Cu(II) with the carbon radical and the subsequent C–N bond formation. Meanwhile, the conflict of the Cu(II)–N intermediate with the electrophilic nitrogen radical results in a higher energy barrier for N–N radical–radical homo-coupling, which uncovers the principle behind the cross-coupling selectivity. We believe this study provides a practical theoretical guide for the design of transition metal-catalyzed radical–radical cross-coupling. On the basis of electrophilicity and nucleophilicity of free radicals, diverse radical–radical cross-coupling could be achieved by the combination of a suitable transition metal with radical species.

## Additional Information

**How to cite this article:** Qi, X. *et al*. Stabilization of Two Radicals with One Metal: A Stepwise Coupling Model for Copper-Catalyzed Radical–Radical Cross-Coupling. *Sci. Rep.* **7**, 43579; doi: 10.1038/srep43579 (2017).

**Publisher's note:** Springer Nature remains neutral with regard to jurisdictional claims in published maps and institutional affiliations.

## Supplementary Material

Supplementary Information

## Figures and Tables

**Figure 1:**
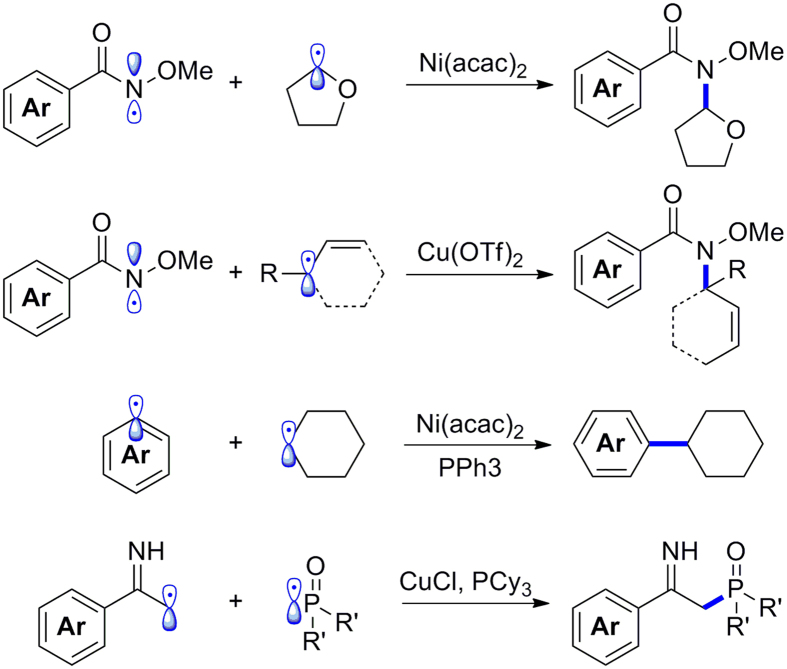
Transition metal-catalyzed radical–radical cross-coupling reactions.

**Figure 2:**
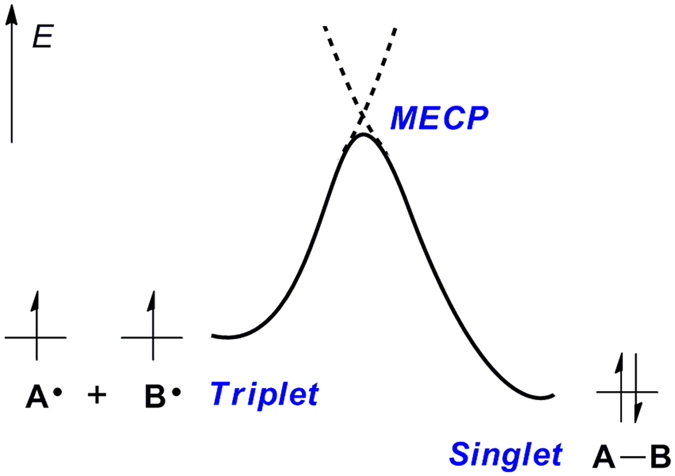
A diagrammatic representation of one possible radical–radical cross-coupling process via the minimum energy crossing point (MECP).

**Figure 3:**
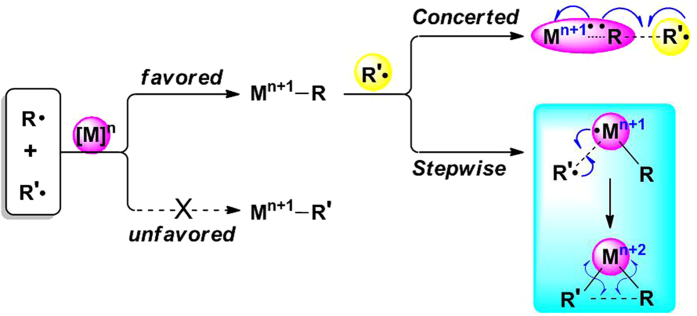
Proposed mechanism for transition metal-catalyzed radical-radical cross-coupling.

**Figure 4:**
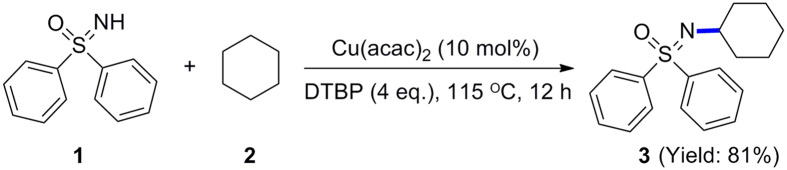
Copper-catalyzed oxidative C(sp3)–H/N–H coupling of sulfoximine with cyclohexane.

**Table 1:** Calculated global electrophilicity *ω*° and global nucleophilicity *N*°, in eV, for carbon radical 7, nitrogen radical 9, and Cu(II) intermediate 15.

Reactivity indices	7 (C·)	9 (N·)	15 (Cu^II^–N)
Global electrophilicity *ω*°	0.99	3.54	3.12
Global nucleophilicity *N*°	3.06	1.14	1.79
