# Supplementary material for: Stabilization of Two Radicals with One Metal: A Stepwise Coupling Model for Copper-Catalyzed Radical–Radical Cross-Coupling
Source: Sci Rep. 2017 Mar 8;7:43579. doi: 10.1038/srep43579 (PMC5341085; doi:10.1038/srep43579)
Supplement: Supplementary Information [file srep43579-s1.doc]

**Supplementary Information**

Stabilization of Two Radicals with One Metal: A Stepwise Coupling Model for Copper-Catalyzed Radical*–*Radical Cross-Coupling

Xiaotian Qi, Lei Zhu, Ruopeng Bai,^*^ and Yu Lan^*^

*School of Chemistry and Chemical Engineering, Chongqing University, Chongqing, 400030 (P. R. China)*

E-mail: ruopeng@cqu.edu.cn

[lanyu@cqu.edu.cn](mailto:lanyu@cqu.edu.cn)

**Table of contents**

1. Complete reference for Gaussian 09 Page S2

2. The generation of *tert*-butoxide anion and a Cu(III) complex Page S2

3. The reduction of Cu(acac)_2_ by carbon radical **7** or nitrogen radical **9**  Page S3

4. Energy difference between **13-sing** and **13-trip** Page S4

5. Energy difference between **18-trip** and **18-sing** Page S4

6. Comparison between the relative free energy of 10-sing and 10’-sing Page S5

7. Computational details for global electrophilicity *ω*° and global nucleophilicity *N*° obtained at B3-LYP/6-31G(d) (SDD basis set for Cu) level of theory Page S6

8. Absolute calculation energies, enthalpies, and free energies Page S6

9. B3LYP geometries for all the compounds and transition states Page S8

### 1. Complete reference for Gaussian 09

Gaussian 09, Revision D.01, Frisch, M. J.; Trucks, G. W.; Schlegel, H. B.; Scuseria, G. E.; Robb, M. A.; Cheeseman, J. R.; Scalmani, G.; Barone, V.; Mennucci, B.; Petersson, G. A.; Nakatsuji, H.; Caricato, M.; Li, X.; Hratchian, H. P.; Izmaylov, A. F.; Bloino, J.; Zheng, G.; Sonnenberg, J. L.; Hada, M.; Ehara, M.; Toyota, K.; Fukuda, R.; Hasegawa, J.; Ishida, M.; Nakajima, T.; Honda, Y.; Kitao, O.; Nakai, H.; Vreven, T.; Montgomery, Jr., J. A.; Peralta, J. E.; Ogliaro, F.; Bearpark, M.; Heyd, J. J.; Brothers, E.; Kudin, K. N.; Staroverov, V. N.; Kobayashi, R.; Normand, J.; Raghavachari, K.; Rendell, A.; Burant, J. C.; Iyengar, S. S.; Tomasi, J.; Cossi, M.; Rega, N.; Millam, N. J.; Klene, M.; Knox, J. E.; Cross, J. B.; Bakken, V.; Adamo, C.; Jaramillo, J.; Gomperts, R.; Stratmann, R. E.; Yazyev, O.; Austin, A. J.; Cammi, R.; Pomelli, C.; Ochterski, J. W.; Martin, R. L.; Morokuma, K.; Zakrzewski, V. G.; Voth, G. A.; Salvador, P.; Dannenberg, J. J.; Dapprich, S.; Daniels, A. D.; Farkas, Ö.; Foresman, J. B.; Ortiz, J. V.; Cioslowski, J.; Fox, D. J. Gaussian, Inc., Wallingford CT, **2013**.

### 2. The generation of *tert*-butoxide anion and a Cu(III) complex

**Figure S1**. Calculated free energy for the formation of *tert*-butoxide anion and a Cu(III) complex **10'**.

The mechanism proposed in the experiment involves the formation of a Cu(III) complex and *tert*-butoxide anion. Besides the combination of a *tert*-butoxyl radical with Cu(acac)_2_, the formation of a Cu(III) complex and *tert*-butoxide anion from **10a-sing** is also considered in calculation. As shown in Figure S1, this process is determined to be endothermic by 77.9 kcal/mol, which is thermodynamically unfavorable. Thus, this mechanism could be safely excluded.

### 3. The reduction of Cu(acac)_2_ by carbon radical 7 or nitrogen radical 9

**Figure S2**. Calculated activation free energy for the reduction of Cu(acac)_2_ using a) carbon radical **7** or b) nitrogen radical **9**.

In this study, the reduction of Cu(acac)_2_ using carbon radical **7** was also calculated. As shown in Figure S2a, activation free energy for the nucleophilic addition of carbon radical **7** to the middle carbon of diketonate via transition state **11-C-ts** is determined to be 26.1 kcal/mol. This value is 2.5 kcal/mol higher than that of **11-ts**, which indicates this possibility for the reduction of Cu(acac)_2_ is unfavorable. Figure S2b shows the transition state for the attack of nitrogen radical **9** to the middle carbon of diketonate; activation free energy for this process is 18.5 kcal/mol. Although this value is lower than that of **11-ts**, *tert*-butoxyl radical is the main radical species at the initial phase of this reaction and the concentration of nitrogen radical would be much lower. Consequently, the reduction of Cu(acac)_2_ is most likely to be accomplished by the *tert*-butoxyl radical.

### 4. Energy difference between 13-sing and 13-trip

**Figure S3**. Energy difference between **13-sing** and the corresponding triplet structure **13-trip**. The values in square brackets are the relative free energies.

As shown in Figure S3, corresponding triplet structure of **13-sing** is located as **13-trip**, the relative free energy of which is 13.2 kcal/mol. Comparison between **13-trip** and **13-sing** suggests that the triplet state structure is 29.1 kcal/mol higher than that of singlet. Consequently, the spin state of Cu(I) complex **13-sing** should be singlet instead of triplet.

### 5. Energy difference between 18-trip and 18-sing

**Figure S4**. Energy difference between **18-trip** and the corresponding singlet structure **18-sing**. The values in square brackets are the relative free energies.

As shown in Figure S4, corresponding singlet structure of **18-trip** is located as **18-sing**, the relative free energy of which is found to be 2.6 kcal/mol higher than that of **18-trip**. Thus, the spin state of Cu(II) complex **18-trip** prefers to be triplet.

### 6. Comparison between the relative free energy of 10-sing and 10’-sing.

**Figure S5**. Comparison between the relative free energy of 10-sing and 10’-sing.

We have also considered displace an oxygen from the coordination sphere of copper in 10-sing using R. As shown in Figure S5, when oxygen of the ligand is replaced by R˙, the relative free energy of generated intermediate (10a’-sing, 10b’-sing, and 10c’-sing) is much higher than that of **10a-sing**, **10b-sing**, and **10c-sing**. It turned out that the combination of acac anion with copper(II) is very tight; it is very difficult to displace an oxygen from the coordination sphere of copper in 10-sing.

### 7. Computational details for global electrophilicity *ω*° and global nucleophilicity *N*° obtained at B3-LYP/6-31G(d) (SDD basis set for Cu) level of theory

|  | *E*^α^_HOMO_ | *E*^β^_HOMO_ | μ° | η° | *ω*° | *N*° |
| --- | --- | --- | --- | --- | --- | --- |
| (CN)_2_CH• | -0.28975 | -0.17648 | -6.340728 | 3.080944 | 6.5247586 | -7.8812 |
| C• | -0.17723 | -0.02981 | -2.815744 | 4.009824 | 0.9886237 | -4.820656 |
| N• | -0.24783 | -0.11873 | -4.985216 | 3.51152 | 3.5386924 | -6.740976 |
| **15** | -0.224 | -0.10571 | -4.484056 | 3.217488 | 3.124605 | -6.092800 |

### 8. Absolute calculation energies, enthalpies, and free energies

| Geometry | E_(elec-B3LYP)_^1^ | E_(solv, M06)_^2^ | G_(corr-B3LYP)_^3^ | H_(corr-B3LYP)_^4^ | IF^5^ |
| --- | --- | --- | --- | --- | --- |
| **1** | -991.944621 | -991.684480 | 0.162505 | 0.216563 | - |
| **2** | -235.876428 | -235.747088 | 0.142468 | 0.177876 | - |
| **3** | -1226.615519 | -1226.232506 | 0.306222 | 0.374525 | - |
| **4** | -466.060179 | -465.868775 | 0.213551 | 0.266720 | - |
| **5** | -233.001933 | -232.906335 | 0.093482 | 0.130523 | - |
| ***t*BuO-** | -233.034489 | -233.015648 | 0.091494 | 0.127734 | - |
| **6-ts** | -468.864709 | -468.643210 | 0.247156 | 0.304302 | -1591.6 |
| **7** | -235.209778 | -235.082052 | 0.126657 | 0.163361 | - |
| **8-ts** | -1224.940110 | -1224.584575 | 0.270193 | 0.343658 | -1618.0 |
| **9** | -991.275416 | -991.007786 | 0.148994 | 0.204252 | - |
| **10** | -887.824772 | -2330.711189 | 0.178604 | 0.246165 | - |
| **10'** | -887.548392 | -2330.453052 | 0.180601 | 0.246414 | - |
| **10a-sing** | -1120.818570 | -2563.617786 | 0.297078 | 0.379557 | - |
| **10a-trip** | -1120.834459 | -2563.628937 | 0.289603 | 0.378549 | - |
| **10b-sing** | -1123.032669 | -2565.810548 | 0.330907 | 0.413050 | - |
| **10b-trip** | -1123.039451 | -2565.807634 | 0.323725 | 0.411589 | - |
| **10c-sing** | -1879.097600 | -3321.731239 | 0.351960 | 0.452587 | - |
| **10c-trip** | -1879.109739 | -3321.738141 | 0.348643 | 0.452204 | - |
| **11-ts** | -1120.817324 | -2563.616270 | 0.293728 | 0.377537 | -393.2 |
| **11-C-ts** | -1123.008484 | -2565.774899 | 0.327294 | 0.409591 | -464.2 |
| **11-N-ts** | -1879.094401 | -3321.725006 | 0.347844 | 0.450689 | -358.1 |
| **12-MECP** | -1120.825161 | -2563.628418 | - | - | - |
| **13-sing** | -1120.860520 | -2563.681362 | 0.295821 | 0.380346 | - |
| **13-trip** | -1120.832059 | -2563.635329 | 0.296219 | 0.379538 | - |
| **14** | -578.2460305 | -578.050458 | 0.197845 | 0.255234 | - |
| **15′** | -2112.204384 | -3554.763100 | 0.470554 | 0.588011 | - |
| **15** | -1533.945983 | -2976.686636 | 0.250428 | 0.329670 | - |
| **16′** | -1356.090663 | -2798.787805 | 0.446615 | 0.547211 | - |
| **16** | -777.832061 | -2220.721034 | 0.226368 | 0.289765 | - |
| **17′** | -1353.925597 | -2796.646864 | 0.414381 | 0.514651 | - |
| **17** | -775.665901 | -2218.573946 | 0.191057 | 0.257148 | - |
| **18-trip** | -1766.969290 | -3209.618432 | 0.364679 | 0.463707 | - |
| **18-sing** | -1766.963803 | -3209.617717 | 0.368076 | 0.464391 |  |
| **19-trip** | -1769.159878 | -3211.781863 | 0.391519 | 0.496141 | - |
| **19-sing** | -1769.179981 | -3211.812092 | 0.401225 | 0.498127 |  |
| **20-MECP** | -1769.149034 | -3211.774870 | - | - | - |
| **21-ts** | -1769.163003 | -3211.799982 | 0.400214 | 0.496744 | -285.5 |
| **22** | -1769.227838 | -3211.870903 | 0.403149 | 0.499803 | - |
| **23-MECP** | -1769.143217 | -3211.771043 | - | - | - |
| **24** | -1534.566001 | -2977.326200 | 0.258750 | 0.342168 | - |
| **25-ts** | -1767.618754 | -3210.275257 | 0.373567 | 0.471566 | -1192.7 |
| **26-trip** | -2525.240588 | -3967.721641 | 0.420840 | 0.536964 | - |
| **26-sing** | -2525.252575 | -3967.742403 | 0.426430 | 0.537563 | - |
| **27-MECP** | -2525.237052 | -3967.724858 | - | - | - |
| **28-ts** | -2525.228640 | -3967.719486 | 0.423148 | 0.535705 | -314.1 |
| **29** | -2525.269747 | -3967.768407 | 0.423811 | 0.537845 | - |
| **30-MECP** | -2525.207240 | -3967.694753 | - | - | - |

^1^The electronic energy calculated by B3LYP in gas phase. ^2^The electronic energy calculated by M06 in cyclohexane solvent. ^3^The thermal correction to Gibbs free energy calculated by B3LYP in gas phase. ^4^The thermal correction to enthalpy calculated by B3LYP in gas phase. ^5^The B3LYP calculated imaginary frequencies for the transition states.

### 9. B3LYP geometries for all the compounds and transition states

**1**

S -0.01255500 1.32433900 -0.09361200

C -1.43273200 0.22517200 -0.01352500

C -1.88328500 -0.14756200 1.25384000

C -2.05838100 -0.22947500 -1.17582500

C -2.97820400 -1.00628700 1.35711500

H -1.39428900 0.24964600 2.13703500

C -3.15466400 -1.08464200 -1.05803100

H -1.70085500 0.10774300 -2.14179400

C -3.61024500 -1.47643500 0.20366000

H -3.34146200 -1.30043700 2.33768800

H -3.65642600 -1.44060300 -1.95346900

H -4.46416300 -2.14313600 0.28775200

C 1.42271700 0.20141500 -0.03207300

C 1.81049700 -0.47105100 -1.19343600

C 2.13058800 0.05358300 1.16200900

C 2.91961800 -1.31647300 -1.15195000

H 1.25681800 -0.31893600 -2.11492100

C 3.24091700 -0.79245200 1.19201200

H 1.81771600 0.60903800 2.03948200

C 3.63193900 -1.47851100 0.03965200

H 3.23044500 -1.84387700 -2.04955000

H 3.80248600 -0.91201900 2.11444200

H 4.49666700 -2.13594400 0.06786000

O 0.05550200 2.08926600 1.17841100

N -0.16516700 1.96909600 -1.49532700

H 0.63350500 2.57762800 -1.68741900

**2**

C -1.46705100 0.03867900 -0.22962900

H -1.53551200 0.04018100 -1.32798000

H -2.49800000 0.06575500 0.14646100

C -0.69990900 1.28931800 0.22946100

H -0.73229100 1.34835800 1.32786000

H -1.19200600 2.19591400 -0.14587900

C 0.76697800 1.25070800 -0.22944400

H 1.30595600 2.13038000 0.14569600

H 0.80220200 1.30774900 -1.32779000

C -0.76690700 -1.25057900 0.22976500

H -1.30610700 -2.13017600 -0.14516900

H -0.80193100 -1.30758600 1.32816500

C 0.69985700 -1.28931200 -0.22973800

H 0.73147600 -1.34776300 -1.32816300

H 1.19195500 -2.19618700 0.14488000

C 1.46718800 -0.03883000 0.22960600

H 2.49828900 -0.06582400 -0.14612300

H 1.53504200 -0.04070400 1.32791400

**3**

S 0.24457400 0.06070300 -0.69415700

C 1.20804200 1.47979600 -0.14835900

C 2.23106100 1.91924500 -0.99016700

C 0.93578300 2.11423400 1.06480900

C 3.00629000 3.01174900 -0.59904400

H 2.39569100 1.42333600 -1.94084600

C 1.71571200 3.20807500 1.44189100

H 0.11208000 1.76358000 1.67546600

C 2.75127600 3.65272800 0.61546800

H 3.80287200 3.36626200 -1.24699400

H 1.51054200 3.71608000 2.38008600

H 3.35542700 4.50463000 0.91550200

C 1.20908700 -1.38398700 -0.11783500

C 1.20625000 -1.70838000 1.24114100

C 1.91587900 -2.15289600 -1.04354100

C 1.93394000 -2.81503400 1.67920900

H 0.63396900 -1.10630600 1.94036500

C 2.63964600 -3.26018400 -0.59566300

H 1.88191900 -1.88310400 -2.09352400

C 2.65104800 -3.58851200 0.76185300

H 1.93784600 -3.07599500 2.73388800

H 3.19165100 -3.86721000 -1.30799400

H 3.21565600 -4.45079800 1.10607000

O 0.33448200 -0.00833900 -2.17941200

N -1.07084900 0.20708100 0.09962600

C -2.09183000 -0.85978100 -0.00874900

H -1.61526000 -1.85043300 -0.08995900

C -3.00370000 -0.65810800 -1.24056800

C -2.93241900 -0.84109600 1.28194900

H -3.66160100 -1.53665400 -1.32432000

H -2.38942200 -0.62781200 -2.14717800

C -3.85912000 0.61274700 -1.12588500

H -2.27027500 -0.94741300 2.15034900

H -3.59220300 -1.72104300 1.27120000

C -3.78367600 0.43201000 1.40352300

H -4.52048000 0.69574600 -1.99805000

H -3.19819600 1.48838100 -1.14209500

C -4.68321200 0.62116100 0.17148400

H -3.11702800 1.29815600 1.50306300

H -4.39101800 0.38858900 2.31726900

H -5.25078700 1.55717600 0.25632500

H -5.42482800 -0.19188100 0.13670700

**4**

O -0.49208300 0.02629900 -0.60212000

O 0.49173400 -0.47163500 0.37460300

C -1.78855700 -0.01703300 0.02647000

C -2.14025100 -1.45923300 0.41722800

C -1.83756100 0.92644000 1.23511900

C -2.70134800 0.48377500 -1.10122500

H -2.07566400 -2.11473600 -0.45772600

H -1.45135000 -1.83387400 1.17861300

H -3.15979500 -1.51104300 0.81627900

H -1.58560200 1.94843100 0.93174800

H -2.84138300 0.93476800 1.67488000

H -1.12619400 0.60758900 2.00095500

H -3.74194800 0.49521600 -0.76016400

H -2.42437700 1.49941400 -1.40261400

H -2.62985100 -0.17011700 -1.97635700

C 1.78834000 0.01121400 -0.02926800

C 2.13901800 -0.51665200 -1.42731300

C 1.83918700 1.54360300 0.02750300

C 2.70138600 -0.61033400 1.03641400

H 2.06308300 -1.60899400 -1.44891100

H 1.45695300 -0.11022800 -2.17860100

H 3.16246400 -0.23324900 -1.69840400

H 1.59428900 1.89671000 1.03519300

H 2.84158200 1.90397300 -0.23025200

H 1.12327200 1.97789200 -0.67465900

H 3.74136300 -0.32282000 0.84897400

H 2.42127600 -0.26416800 2.03679200

H 2.63339300 -1.70275600 1.01482500

**5**

O -0.00180700 -0.26266500 1.43192800

C 0.00021500 0.02631900 0.08208300

C -1.27358400 0.79952900 -0.31557300

C 1.28224000 0.78594400 -0.31510000

C -0.00733700 -1.38832100 -0.57968200

H -2.16496200 0.23916100 -0.01681200

H -1.29600600 1.77201100 0.18831800

H -1.31029300 0.97459300 -1.39699600

H 2.16755700 0.21536700 -0.01757000

H 1.32057200 0.96216500 -1.39628300

H 1.31554000 1.75737100 0.19019300

H -0.00566300 -1.26075600 -1.66765900

H 0.87980100 -1.95475900 -0.28379700

H -0.90128800 -1.94466200 -0.28518200

***t*BuO-**

O 1.65354439 -0.03408913 -2.12835777

C 1.17620058 1.31388820 -2.12835777

C 1.68916906 2.04010915 -0.87095312

H 2.75577462 2.11457825 -0.91226828

H 1.26454207 3.02128749 -0.82758270

H 1.40360350 1.48904357 0.00064256

C 1.68916958 2.04010856 -3.38576255

H 1.35874454 1.51766214 -4.25912905

H 1.30648670 3.03920852 -3.40167143

H 2.75869093 2.06803649 -3.37013817

C -0.36379923 1.31311418 -2.12835759

H -0.72097256 2.32172970 -2.13304077

H -0.72021008 0.80448150 -2.99965782

H -0.72021445 0.81259351 -1.25237400

**6-ts**

C 1.48354900 1.26840300 0.20139900

H 0.97598700 2.15209900 -0.20295200

H 1.40673400 1.32871200 1.29611700

C 1.49583100 -1.27588700 0.20449300

H 0.99638300 -2.16523000 -0.19752800

H 1.41970400 -1.33418400 1.29936600

C 0.81326500 -0.00755400 -0.27743100

H 0.62415800 -0.00992000 -1.35837600

H -0.36192600 -0.01429400 0.25605100

O -1.43079000 -0.02384200 0.88866800

C -2.53904700 -0.00014600 0.00674700

C -2.54400500 1.28593200 -0.83930800

H -3.44904500 1.35805800 -1.45445100

H -2.49430100 2.16489500 -0.18799100

H -1.67893600 1.31033000 -1.51270000

C -2.55846000 -1.25199200 -0.88908000

H -2.51848500 -2.15613200 -0.27249600

H -3.46426900 -1.28999700 -1.50613000

H -1.69370300 -1.25992600 -1.56323900

C -3.75708100 -0.01182000 0.95545700

H -4.68857100 0.00639900 0.37764200

H -3.74614000 -0.91259300 1.57715800

H -3.73426300 0.86214700 1.61401100

C 3.69326300 0.00678500 0.29341300

H 3.71438200 0.00818300 1.39318000

H 4.73947900 0.01148200 -0.03891600

C 2.97715500 1.27376100 -0.19990800

H 3.46497700 2.17209600 0.20006800

H 3.05622500 1.33294200 -1.29510700

C 2.98939700 -1.26818300 -0.19686000

H 3.48570500 -2.16085400 0.20534100

H 3.06902600 -1.32926700 -1.29191500

**7**

C 0.00000100 1.46432700 -0.16717000

H 0.00000000 2.52945100 -0.38628000

C 1.29099000 0.77858300 0.15705800

H 1.48386800 0.83616200 1.24734900

H 2.13609200 1.29048600 -0.32062500

C 1.26842500 -0.71304000 -0.24270200

H 2.16559900 -1.21659400 0.13932800

H 1.30148800 -0.79095500 -1.33842400

C -1.29098900 0.77858500 0.15706000

H -2.13609000 1.29049000 -0.32062200

H -1.48386500 0.83616100 1.24735000

C -1.26842600 -0.71303900 -0.24270300

H -1.30148700 -0.79095000 -1.33842500

H -2.16560100 -1.21659200 0.13932400

C -0.00000100 -1.41158100 0.27077600

H -0.00000100 -2.46602100 -0.03442500

H -0.00000200 -1.40464700 1.37153300

**8-ts**

S 0.33926900 -0.39712500 0.39683100

C 2.04465600 -0.87042600 0.02393700

C 2.87704900 -1.15761600 1.10562800

C 2.49720600 -0.95381500 -1.29515100

C 4.20100300 -1.52489200 0.85754800

H 2.48057600 -1.10215700 2.11369800

C 3.82186800 -1.32251100 -1.52769300

H 1.81988800 -0.75303800 -2.11737000

C 4.67235700 -1.60441300 -0.45449300

H 4.86089800 -1.75241800 1.68990000

H 4.18812700 -1.39538000 -2.54776300

H 5.70311700 -1.89179900 -0.64294600

C 0.27607800 1.39829800 0.12457200

C -0.01347400 1.88805100 -1.14961600

C 0.51053100 2.25518400 1.20173900

C -0.05389500 3.26854600 -1.34962100

H -0.22652400 1.19786800 -1.95900200

C 0.47001200 3.63345500 0.98837900

H 0.70188900 1.84138600 2.18573500

C 0.19170600 4.13849500 -0.28456800

H -0.28604400 3.66306000 -2.33476200

H 0.64870500 4.31219900 1.81763600

H 0.15800100 5.21268300 -0.44485200

O 0.13576800 -0.58377400 1.85716700

N -0.44827500 -1.17512800 -0.70261900

H -1.59753900 -0.77993400 -0.88281800

O -2.65022000 -0.24157500 -0.73545800

C -3.60545700 -1.06310300 -0.08333900

C -4.95470400 -0.40718100 -0.46413800

H -5.77695500 -0.95416900 0.01124100

H -4.98122800 0.63345500 -0.12645300

H -5.09779800 -0.42633000 -1.54898800

C -3.54584400 -2.50588900 -0.60992000

H -2.57767600 -2.95804200 -0.37178500

H -4.33415400 -3.12084700 -0.16041600

H -3.67332500 -2.51951900 -1.69803500

C -3.40819700 -1.01200000 1.44136700

H -4.18529800 -1.59060900 1.95523800

H -2.43032600 -1.41880500 1.71623400

H -3.45447300 0.02399100 1.79309100

**9**

S -0.00025200 1.11411900 -0.66286800

C -1.46207100 0.17787500 -0.13089600

C -2.16653100 0.64626700 0.97925000

C -1.86865000 -0.95794500 -0.83521500

C -3.29573000 -0.05545400 1.40379900

H -1.83080300 1.54655900 1.48190800

C -2.99997300 -1.64862700 -0.39979800

H -1.32064900 -1.27706600 -1.71453600

C -3.70907100 -1.20042200 0.71800300

H -3.85413200 0.29554700 2.26693600

H -3.33131800 -2.53193000 -0.93823000

H -4.59040300 -1.74147400 1.05129900

C 1.46244900 0.17954000 -0.13021100

C 1.87458400 -0.95111400 -0.83957100

C 2.16167700 0.64322000 0.98525600

C 3.00607700 -1.64148400 -0.40401300

H 1.33066400 -1.26621700 -1.72289200

C 3.29095100 -0.05822000 1.41001300

H 1.82218700 1.53994400 1.49173500

C 3.70974600 -1.19815300 0.71911500

H 3.34174600 -2.52070300 -0.94644000

H 3.84525300 0.28912200 2.27726600

H 4.59115700 -1.73896100 1.05260300

O -0.00053000 2.36926100 0.13317700

N -0.00231000 1.00175500 -2.23050900

**10**

C 2.60783900 -1.23721500 0.00015300

C 3.41219900 -2.51967400 0.00039000

H 3.14980900 -3.11257000 -0.88313700

H 4.48976000 -2.33811400 0.00519000

H 3.14237200 -3.11703300 0.87859300

C 3.27030800 -0.00000700 0.00139200

H 4.35354800 -0.00001000 0.00291500

C 2.60784700 1.23720900 0.00027200

C 3.41221900 2.51966000 0.00140900

H 3.14685700 3.11351000 0.88339900

H 4.48978700 2.33808700 0.00037800

H 3.14536000 3.11607400 -0.87835000

C -2.60784000 1.23721600 0.00029200

C -3.41219200 2.51968000 0.00028100

H -4.48976200 2.33812300 0.00202500

H -3.14472500 3.11568400 0.88014300

H -3.14742100 3.11393300 -0.88160600

C -3.27031500 0.00001000 0.00059900

H -4.35355600 0.00001600 0.00100800

C -2.60785300 -1.23720600 0.00042800

C -3.41222600 -2.51965700 0.00109300

H -3.14674600 -3.11391500 0.88276100

H -4.48979400 -2.33808300 0.00022600

H -3.14548600 -3.11566400 -0.87898800

O -1.34017200 1.39908000 -0.00013300

O -1.34019000 -1.39908600 -0.00013900

O 1.34017300 -1.39908700 -0.00156200

O 1.34018400 1.39909100 -0.00154500

Cu 0.00000400 -0.00000400 -0.00087500

**10'**

C -2.53997100 1.22019000 0.00013400

C -3.28498800 2.53207900 0.00002900

H -2.99580000 3.11261000 -0.88269700

H -4.36486900 2.37634400 -0.00025900

H -2.99629400 3.11245900 0.88302700

C -3.21795100 0.00002700 0.00038500

H -4.29914800 0.00004400 0.00070600

C -2.53999200 -1.22017600 0.00004700

C -3.28507100 -2.53202100 0.00016000

H -2.99460600 -3.11336000 0.88192200

H -4.36493900 -2.37622000 0.00213100

H -2.99771300 -3.11162100 -0.88379300

C 2.53998800 -1.22017700 0.00003700

C 3.28503900 -2.53204900 0.00024100

H 4.36491800 -2.37629400 -0.00037400

H 2.99658300 -3.11207200 0.88355400

H 2.99563200 -3.11294700 -0.88216900

C 3.21792600 -0.00000400 0.00010900

H 4.29912300 0.00000800 0.00018400

C 2.53995400 1.22020200 0.00010000

C 3.28505800 2.53203800 0.00000700

H 2.99566000 3.11287600 0.88246200

H 4.36492500 2.37622600 0.00062800

H 2.99663600 3.11215700 -0.88325400

O 1.25782900 -1.35522500 -0.00009600

O 1.25784700 1.35518600 0.00010600

O -1.25782400 1.35516800 -0.00000200

O -1.25788000 -1.35516700 -0.00053100

Cu 0.00000600 -0.00002000 -0.00018500

**10a-sing**

C 2.29999500 -1.81291800 0.17685400

C 2.78262700 -3.15965500 0.67276700

H 2.57040300 -3.92224600 -0.08451500

H 3.85223100 -3.15689600 0.89444200

H 2.22405300 -3.43211100 1.57467700

C 3.20686600 -0.75815500 0.02880800

H 4.24266900 -0.92935400 0.29227300

C 2.85126900 0.48233600 -0.52030100

C 3.91222500 1.53557000 -0.75874100

H 3.63043200 2.45583800 -0.23578700

H 4.89944100 1.21240700 -0.42059100

H 3.95579400 1.77054300 -1.82811700

C -1.08089500 2.34588200 -0.65818700

C -1.66436100 3.39387500 -1.58852900

H -1.83597900 4.34345300 -1.07597400

H -0.98215000 3.55344000 -2.43081100

H -2.61113200 3.02923900 -2.00189500

C -0.83155400 2.67384300 0.66759100

H -1.06717300 3.68933200 0.96358400

C -0.33490400 1.79673800 1.68074300

C -0.19936700 2.34376500 3.09447600

H 0.82085800 2.16741400 3.45255200

H -0.42861600 3.41035900 3.16801300

H -0.87250700 1.78955300 3.75924600

O -0.90590000 1.21527900 -1.26335000

O -0.01673700 0.59756400 1.49837600

O 1.04929600 -1.78337800 -0.09378800

O 1.67488100 0.82215700 -0.87537900

O -1.35675900 -1.32575500 -0.92998400

Cu 0.08078600 -0.23054900 -0.58634400

C -2.37913200 -1.63469000 -0.01548400

C -3.28881700 -0.42527000 0.26691500

H -2.73996900 0.35390600 0.80352700

H -3.65784700 -0.00228400 -0.67291400

H -4.14846200 -0.72080100 0.88057000

C -3.18796000 -2.71776100 -0.78541200

H -4.03536200 -3.04425300 -0.17074600

H -3.56642800 -2.31105600 -1.72772400

H -2.55189800 -3.58018400 -1.00413400

C -1.83551100 -2.23689000 1.29332700

H -1.19508000 -3.09760700 1.07749600

H -1.24539200 -1.49483500 1.83879600

H -2.66180400 -2.56489000 1.93603000

**10a-trip**

C 2.49145500 0.22559300 1.39584900

C 3.34295000 -0.47901500 2.43283600

H 3.28457700 -1.56296300 2.28079200

H 4.38998300 -0.16776500 2.39693500

H 2.93998400 -0.27413500 3.43108300

C 3.08361800 1.15551200 0.53049200

H 4.14767200 1.33567400 0.62609100

C 2.37569000 1.87620200 -0.45004300

C 3.11724600 2.85523800 -1.33715900

H 2.66680600 3.84887000 -1.23568900

H 4.18213100 2.91533400 -1.09863600

H 2.99806300 2.55458800 -2.38426800

C -2.80611300 1.33532800 -0.63098500

C -3.67099600 2.10732600 -1.60673700

H -4.73594000 1.89474500 -1.48322200

H -3.49991900 3.18131100 -1.47098800

H -3.36872800 1.86128400 -2.63078900

C -3.41345500 0.48495200 0.30923400

H -4.49393900 0.40540300 0.30045700

C -2.70659700 -0.23255900 1.28642500

C -3.46828000 -1.06849500 2.29544600

H -3.26687300 -0.69043000 3.30444700

H -4.54685100 -1.05978400 2.11897200

H -3.10369200 -2.10125700 2.26222600

O -1.55371600 1.54214600 -0.74455600

O -1.43798700 -0.24621800 1.42829400

O 1.25563700 -0.10470000 1.40271500

O 1.12576800 1.78765400 -0.67388000

O 0.06203200 -1.16216200 -1.25858900

Cu -0.15001500 0.64819900 0.26800900

C 0.52746300 -2.46422100 -1.20238400

C -0.23038500 -3.14330900 -2.39104100

H -1.31080900 -3.09524000 -2.23240300

H 0.01688700 -2.65228800 -3.33595000

H 0.08151600 -4.19245400 -2.43723500

C 2.04633100 -2.51512700 -1.45761800

H 2.40673600 -3.55004500 -1.48061100

H 2.28747800 -2.03635000 -2.41202500

H 2.57777000 -1.98146400 -0.66365400

C 0.13799700 -3.14483800 0.12336000

H 0.59000300 -2.60706300 0.96123400

H -0.94788600 -3.12649500 0.25537600

H 0.48005800 -4.18650900 0.13766500

**10b-sing**

C -1.14416200 2.10092100 -0.80483000

C -1.90216800 2.80398400 -1.91514900

H -2.88901000 2.34402900 -2.04347500

H -2.02931900 3.87163800 -1.71931600

H -1.36121000 2.67403100 -2.85953700

C -0.69078600 2.84600300 0.27857900

H -0.91481400 3.90659200 0.25497700

C 0.04959300 2.37171000 1.40561800

C 0.47599300 3.39260500 2.45211100

H 1.54938700 3.59145500 2.33963100

H -0.06114600 4.34179100 2.36822900

H 0.32774400 2.97283400 3.45194800

C 2.57859900 -1.77177800 0.38615600

C 3.19397800 -2.96965900 1.07973500

H 4.27828300 -3.01192700 0.95204900

H 2.95865800 -2.93036900 2.14941000

H 2.74964100 -3.89046600 0.68458100

C 3.39539200 -0.88761100 -0.31526600

H 4.45932200 -1.09070300 -0.33168300

C 2.92158100 0.22829000 -1.04459000

C 3.90996100 1.07102800 -1.82795100

H 3.84938800 2.10953300 -1.48374700

H 4.93944800 0.71781800 -1.72771700

H 3.62916700 1.06611200 -2.88726300

O 1.29847900 -1.70487000 0.51722600

O 1.71133100 0.58876400 -1.11497400

O -1.02260900 0.82908700 -1.01686500

O 0.39095900 1.17985500 1.59801900

Cu 0.20175400 -0.26424300 -0.08902800

C -1.29979000 -1.28195900 0.84973200

C -1.54017000 -2.55366500 0.06173600

H -1.91545500 -3.29144600 0.79740300

H -0.59512300 -2.96713200 -0.30369600

C -2.58376000 -2.41096300 -1.05507700

H -2.76650100 -3.39286300 -1.50922900

H -2.18969300 -1.76041000 -1.84684300

C -3.88929500 -1.81836100 -0.50557300

H -4.30945100 -2.50521100 0.24546200

H -4.63717800 -1.73329600 -1.30392200

C -3.63792200 -0.44179100 0.12655200

H -4.56092200 -0.05287600 0.57531700

H -3.34141700 0.25670800 -0.66037000

C -2.53724200 -0.48273600 1.20106900

H -2.26371500 0.52457500 1.52883400

H -2.94293100 -0.98400500 2.10110800

H -0.63810100 -1.42215300 1.70530900

**10b-trip**

C -2.61178600 0.16126400 -1.37058500

C -3.42451300 -0.61070500 -2.38885400

H -3.23355900 -1.68323100 -2.26827300

H -4.49746000 -0.42497300 -2.29621700

H -3.09536400 -0.33762600 -3.39759400

C -3.26857900 0.96980200 -0.42879600

H -4.35042000 1.01562900 -0.46285200

C -2.60535500 1.73366500 0.54564800

C -3.41052800 2.58273500 1.50725200

H -3.11083500 3.63164200 1.40265200

H -4.48734600 2.49798000 1.34123400

H -3.17873800 2.28454500 2.53596300

C 2.60682200 1.73269000 0.54532500

C 3.41215400 2.58236700 1.50624800

H 4.48903500 2.49582400 1.34154700

H 3.11408500 3.63151300 1.39935900

H 3.17881200 2.28645000 2.53525900

C 3.26992400 0.96706000 -0.42777100

H 4.35183900 1.01167800 -0.46107900

C 2.61291400 0.15839100 -1.36932100

C 3.42553500 -0.61538400 -2.38631400

H 3.09770900 -0.34253000 -3.39554900

H 4.49865700 -0.43107400 -2.29282200

H 3.23297300 -1.68755500 -2.26518000

O 1.34179300 1.79040100 0.70788200

O 1.34842200 0.00000100 -1.45902500

O -1.34740000 0.00138300 -1.45938700

O -1.34038400 1.79010600 0.70897600

Cu 0.00060000 0.84238900 -0.33616100

C -0.00220600 -1.32375200 1.78040000

C 1.28968400 -2.07847600 1.67609900

H 1.48614400 -2.59617800 2.63718100

H 2.13103200 -1.38851000 1.53380400

C 1.26431700 -3.13800900 0.55551000

H 2.16349500 -3.76494700 0.61515600

H 1.28865600 -2.62550500 -0.41506500

C -0.00156700 -4.00481500 0.63022500

H -0.00234900 -4.57444900 1.57249700

H -0.00102200 -4.74356800 -0.18171900

C -1.26717000 -3.13775700 0.55359200

H -2.16656300 -3.76451900 0.61180700

H -1.28989600 -2.62515300 -0.41696900

C -1.29402800 -2.07831600 1.67423400

H -2.13506100 -1.38821700 1.53079200

H -1.49188400 -2.59608600 2.63499100

H -0.00251800 -0.37010500 2.30482200

**10c-sing**

C 1.94934900 -2.43187600 -0.79304200

C 1.29582600 -3.79184400 -0.87518600

H 0.44747400 -3.73053400 -1.56551000

H 1.99309800 -4.56290400 -1.21098800

H 0.89530100 -4.06482000 0.10722000

C 3.29581600 -2.26896500 -1.10579100

H 3.86735000 -3.14515000 -1.38460100

C 3.96494700 -1.03054600 -1.01332900

C 5.46255000 -0.97007100 -1.22905700

H 5.94434000 -0.64935500 -0.29833700

H 5.87783200 -1.93356600 -1.53411400

H 5.69006400 -0.21520900 -1.98888000

C 1.63035300 3.13177900 0.06308300

C 1.55993200 4.48531500 -0.61915100

H 1.50595600 5.30672800 0.10008000

H 2.43912900 4.62128300 -1.25802800

H 0.67781200 4.51349600 -1.26919400

C 1.50486900 3.05536100 1.44735900

H 1.36551300 3.99254800 1.97411200

C 1.55872300 1.86467600 2.23175800

C 1.46087000 2.00738300 3.74595000

H 2.26762100 1.43392600 4.21438900

H 1.51099200 3.04619200 4.08420500

H 0.51405800 1.57322500 4.09124000

O 1.82472700 2.16691500 -0.76663300

O 1.67714400 0.70227400 1.77422700

O 1.14453600 -1.50713700 -0.39702600

O 3.41245700 0.07958700 -0.74489200

Cu 1.49563800 0.32874600 -0.44406600

S -1.39433100 -0.40599900 -0.83581700

C -1.68874800 -1.50417000 0.59549600

C -2.48330500 -2.63845000 0.41500300

C -1.14761900 -1.18145200 1.83881500

C -2.75351500 -3.45739200 1.51206600

H -2.86849800 -2.87698700 -0.57076000

C -1.41816700 -2.01481800 2.92702100

H -0.48940600 -0.32451700 1.94079600

C -2.22362800 -3.14449500 2.76725700

H -3.37236900 -4.34161900 1.38529100

H -0.99011900 -1.78276600 3.89841000

H -2.43311000 -3.78643000 3.61883100

C -2.89827700 0.60975200 -0.86459700

C -3.14311300 1.56593400 0.12384800

C -3.78744900 0.39577000 -1.91681000

C -4.31890400 2.31225100 0.05760100

H -2.41951900 1.72954800 0.91436400

C -4.96008500 1.15257300 -1.97136900

H -3.55059900 -0.34676900 -2.67122500

C -5.22602600 2.10595400 -0.98663100

H -4.52425700 3.06010200 0.81845100

H -5.66286000 0.99685500 -2.78516500

H -6.13938900 2.69275800 -1.03343300

N -0.34377600 0.72320800 -0.58781900

O -1.43518900 -1.29440700 -2.02982700

**10c-trip**

C 1.10058200 -2.88420200 0.09700500

C 0.32718200 -3.95071700 0.84238400

H -0.74429700 -3.74163700 0.73851000

H 0.52664200 -4.95380100 0.45630400

H 0.57161300 -3.90974200 1.90844100

C 1.71330300 -3.20318300 -1.12127000

H 1.62780200 -4.22199400 -1.47964000

C 2.39366100 -2.27470900 -1.92958900

C 2.98438500 -2.72779400 -3.24944500

H 4.04905900 -2.47149100 -3.27922600

H 2.86435000 -3.80118500 -3.41673300

H 2.49814800 -2.18099900 -4.06556800

C 3.19720600 2.50687000 -0.09951000

C 3.92810600 3.56520100 -0.90186300

H 4.16806100 4.45185800 -0.30939000

H 4.85351100 3.13872900 -1.30503000

H 3.31042800 3.85858000 -1.75810800

C 2.91616900 2.74166400 1.26022400

H 3.23987600 3.68143400 1.69157300

C 2.26034100 1.82556600 2.09334600

C 2.03727700 2.17849600 3.55195100

H 2.45175100 1.38640600 4.18527800

H 2.49091400 3.13435100 3.82621200

H 0.96098100 2.22601500 3.75774500

O 2.89885200 1.45370100 -0.74543500

O 1.81200000 0.68092400 1.74369400

O 1.11077900 -1.73377300 0.66097500

O 2.56753900 -1.04377800 -1.65692300

Cu 1.92122000 -0.10191400 -0.06732500

S -1.51954700 -0.32818500 -0.59356900

C -2.13410500 -0.64789300 1.08575600

C -3.24849300 -1.47750200 1.24094000

C -1.48615500 -0.07246000 2.17748100

C -3.73904800 -1.71106900 2.52518500

H -3.71092700 -1.93610300 0.37322700

C -1.98142100 -0.32818700 3.45866000

H -0.59025900 0.52014300 2.02582200

C -3.10719800 -1.13529300 3.63194100

H -4.60853400 -2.34793300 2.66226100

H -1.47810800 0.09843500 4.32175700

H -3.48921400 -1.32544700 4.63133500

C -2.64734100 0.91177200 -1.28431200

C -2.72643800 2.19520100 -0.73695500

C -3.40703700 0.53237700 -2.39061600

C -3.60485900 3.11340200 -1.31069500

H -2.10878000 2.46640500 0.11201500

C -4.28045900 1.46392400 -2.95511600

H -3.30450300 -0.47133400 -2.78931600

C -4.37957600 2.74864800 -2.41631600

H -3.68058600 4.11530600 -0.89797000

H -4.88079200 1.18511200 -3.81645500

H -5.06009500 3.47005600 -2.86018200

N -0.19433500 0.50544600 -0.56893100

O -1.72614700 -1.59751100 -1.33930400

**11-ts**

C 3.03022500 -0.90438100 0.74682300

C 3.89695000 -1.69094800 1.70761000

H 4.00706600 -2.71705700 1.33878300

H 4.88704300 -1.24751600 1.83892700

H 3.39593400 -1.75172500 2.68028700

C 3.54521500 0.25078000 0.13482500

H 4.56052700 0.54228100 0.37529200

C 2.83388400 1.04133200 -0.78152100

C 3.50142900 2.25123200 -1.40135600

H 2.89944900 3.14366600 -1.19673300

H 4.51662500 2.40724500 -1.02823400

H 3.53293300 2.12847000 -2.49004800

C -2.02915200 -0.04615600 -1.54224200

C -3.06636900 0.53460100 -2.47056600

H -3.15966600 -0.09567100 -3.36379900

H -4.04892300 0.61898800 -1.99771500

H -2.74498200 1.52592400 -2.81205300

C -2.37471800 -0.51059000 -0.21422700

H -3.39182900 -0.30601700 0.10886900

C -1.89080800 -1.85386500 0.09072900

C -2.82100600 -2.87069900 0.70141200

H -3.08922300 -2.55411700 1.71864000

H -3.75277400 -2.96248400 0.13197500

H -2.32477500 -3.84202300 0.75208900

O -0.83649800 -0.14773500 -1.97150300

O -0.69827800 -2.17840500 -0.15387800

O 1.86007100 -1.38253300 0.56659600

O 1.63271400 0.83590000 -1.16721300

O -1.21164900 0.34431400 0.88357000

C -1.55038000 1.61451600 1.43349700

C -2.74251900 1.47523900 2.39809800

H -2.95369200 2.42313300 2.90762800

H -3.65417800 1.17863100 1.86563600

H -2.52632200 0.71511800 3.15660300

C -0.29531200 2.04253400 2.21884800

H -0.05633600 1.29853200 2.98568000

H 0.56222000 2.12880300 1.54373400

H -0.45364800 3.01162300 2.70829500

C -1.85460200 2.65531800 0.34074000

H -2.77222300 2.40479600 -0.20423300

H -1.99460400 3.65160600 0.77788400

H -1.02892700 2.70423900 -0.37691700

Cu 0.50077300 -0.62456000 -0.58128200

**11-C-ts**

C -3.48221800 0.93456200 1.16168900

C -4.16444000 2.02979000 1.95327700

H -4.41676100 2.85836400 1.28190600

H -5.07165700 1.68251900 2.45384500

H -3.46752900 2.42442900 2.70131400

C -4.03480600 -0.35546200 1.13861700

H -4.94691100 -0.52982700 1.69640900

C -3.47345200 -1.43325100 0.43622800

C -4.14686500 -2.78843600 0.47696200

H -3.44476800 -3.53048900 0.87365200

H -5.05349000 -2.78784000 1.08703800

H -4.39817000 -3.09872900 -0.54360200

C 1.04659400 -0.64946200 -1.99671200

C 1.69327100 -1.58710400 -2.98973300

H 1.58272200 -2.62263600 -2.64716400

H 1.17624400 -1.51604900 -3.95567500

H 2.75359800 -1.36979400 -3.14002400

C 1.78587800 0.45413700 -1.44505500

H 2.77719900 0.58596200 -1.86985300

C 1.04205900 1.67403000 -1.28205600

C 1.68513300 3.01162100 -1.56557000

H 1.64765200 3.63810600 -0.66593400

H 2.72439500 2.91658300 -1.88955300

H 1.11457100 3.53789600 -2.34127900

O -0.17216800 -0.90484900 -1.70920300

O -0.17739500 1.71856300 -0.90130200

O -2.41545300 1.29543200 0.55686400

O -2.40555000 -1.38615900 -0.26471000

C 2.42693800 -0.13819500 0.48417500

C 3.19067200 -1.43445400 0.33581700

C 3.24772900 0.99302600 1.06027500

C 3.83428600 -1.85493700 1.67807000

H 2.53930900 -2.23639500 -0.03431200

H 4.05522500 1.26116400 0.35860200

H 3.03851400 -2.11130600 2.39214300

H 3.99539800 -1.30916700 -0.40782400

H 4.43924900 -2.76179200 1.54086700

H 2.63667700 1.89393500 1.19923900

H 1.44958800 -0.25820300 0.96154700

Cu -1.29329100 0.17189100 -0.54945600

C 3.89377600 0.58263800 2.40461600

H 3.10241800 0.44287400 3.15505800

H 4.54118500 1.38914600 2.77556900

C 4.69424500 -0.72193600 2.26087200

H 5.55475500 -0.54520300 1.59757000

H 5.10773700 -1.02170200 3.23264900

**11-N-ts**

C 3.46203000 0.51057800 1.76423500

C 3.81441200 0.90307000 3.18479800

H 4.23766100 0.03578300 3.70449100

H 4.52853100 1.72930600 3.22727900

H 2.90239100 1.18410400 3.72344100

C 4.08028600 1.17000500 0.69107900

H 4.79167200 1.95497700 0.91837600

C 3.84484000 0.86634900 -0.66131600

C 4.57165300 1.64356800 -1.73896200

H 3.83682800 2.13980300 -2.38304700

H 5.25422700 2.39186100 -1.32848500

H 5.13434700 0.94767600 -2.37123000

C 0.17679000 -2.46587200 -1.69878600

C -0.42228200 -2.86992100 -3.01898700

H 0.37110300 -3.08147000 -3.73903900

H -1.07995800 -3.74094800 -2.92364100

H -1.03057400 -2.03220200 -3.38896200

C -0.67664100 -2.33068900 -0.52473000

H -1.73017800 -2.54213400 -0.69956400

C -0.12673600 -2.90758100 0.68915200

C -0.97799300 -3.79700300 1.56050100

H -1.90347800 -3.27694000 1.84198300

H -1.27062500 -4.71477600 1.03535400

H -0.42479800 -4.06513200 2.46327100

O 1.40943300 -2.19484800 -1.63652800

O 1.06372700 -2.65408600 1.04033700

O 2.60024100 -0.42800200 1.66665400

O 3.05125500 -0.03433500 -1.09270400

Cu 1.95908700 -1.19867300 0.00528400

S -1.56307500 0.36334900 -0.75479900

C -3.11918600 0.16658200 0.18467300

C -4.34605700 0.27465200 -0.47243700

C -3.05289400 -0.11716400 1.55095500

C -5.52520200 0.10082800 0.25566700

H -4.36320200 0.47630700 -1.53811200

C -4.23676800 -0.28548100 2.27097000

H -2.08406100 -0.21635800 2.03120400

C -5.47105100 -0.17512200 1.62410200

H -6.48559400 0.17806000 -0.24686900

H -4.19592500 -0.50541900 3.33435600

H -6.39111800 -0.30869900 2.18671500

C -1.09977000 2.09023100 -0.44113100

C -0.31849600 2.41823500 0.66868500

C -1.52351600 3.05989100 -1.35047700

C 0.03145300 3.75335200 0.87419500

H 0.02485100 1.63615900 1.33767400

C -1.16606200 4.39193200 -1.13316100

H -2.10574800 2.76391500 -2.21663200

C -0.39420500 4.73824600 -0.02170500

H 0.64273700 4.02311200 1.73094500

H -1.48622700 5.15699700 -1.83517800

H -0.11736800 5.77607500 0.14313600

O -1.98334400 0.27577700 -2.18539300

N -0.44827700 -0.50147200 -0.13811100

**12-MECP**

C -3.36979700 -0.87498000 -0.52050300

C -4.47101400 -1.69542900 -1.16759300

H -4.57645300 -2.64507500 -0.63038500

H -5.43519900 -1.18015500 -1.17399600

H -4.18698900 -1.94040800 -2.19747500

C -3.67821300 0.40155000 -0.01070200

H -4.69879900 0.74901100 -0.12074000

C -2.76582500 1.26493400 0.61894200

C -3.24032800 2.63637400 1.06484900

H -2.66628900 3.40638600 0.53546600

H -4.30515700 2.79643200 0.87559100

H -3.03968800 2.76601900 2.13412000

C 2.05729900 -0.47246400 1.34165800

C 3.19050400 -0.79586800 2.28362400

H 3.64427500 -1.77158500 2.05685500

H 3.99295600 -0.04902100 2.21073500

H 2.81614800 -0.82081600 3.30964900

C 2.29533500 -0.45811100 -0.15527600

H 3.35955800 -0.35624700 -0.39790900

C 1.79273800 -1.84596000 -0.53284000

C 2.76377200 -2.89461500 -0.99834000

H 3.21220900 -2.60550300 -1.95881900

H 3.59353400 -3.02134900 -0.28909300

H 2.24725900 -3.84959000 -1.11548100

O 0.87370300 -0.37069700 1.75426000

O 0.57730600 -2.12317200 -0.36987300

O -2.22350200 -1.43055100 -0.49905100

O -1.53614900 1.01277300 0.85796900

O 1.47827400 0.46499500 -0.86820400

C 1.84143000 1.87809000 -0.92067600

C 3.23613200 2.04099100 -1.54189200

H 3.43591200 3.10039100 -1.73456400

H 4.03249200 1.67922500 -0.88140700

H 3.29661700 1.50177000 -2.49380600

C 0.77306700 2.46535300 -1.84926900

H 0.80062300 1.96925300 -2.82481300

H -0.22093500 2.32319000 -1.41463500

H 0.94249700 3.53746700 -1.99707800

C 1.75934100 2.53720800 0.46270900

H 2.51814800 2.14460300 1.14844300

H 1.93242600 3.61619500 0.36889700

H 0.77223300 2.37460100 0.90409900

Cu -0.61831300 -0.66462100 0.35301400

**13-sing**

C -2.81973500 -1.46699800 -0.50256000

C -3.37333300 -2.68722900 -1.22167900

H -3.36563500 -3.54436100 -0.53829900

H -4.38980200 -2.53864300 -1.59596400

H -2.71866200 -2.94364500 -2.06316200

C -3.63557800 -0.31803600 -0.40734100

H -4.61954700 -0.38445200 -0.85825200

C -3.30981100 0.90595800 0.20695300

C -4.33817300 2.02416600 0.18633000

H -3.90827900 2.90527500 -0.30462300

H -5.26119900 1.74464800 -0.32851300

H -4.57721800 2.31695800 1.21538700

C 2.07715500 -0.59098500 1.35860800

C 3.21740500 -1.09274900 2.19495400

H 3.94120100 -0.28212700 2.35723600

H 2.85213300 -1.44723800 3.16094900

H 3.74519100 -1.88927800 1.65852400

C 2.35191300 -0.29070000 -0.12356400

H 3.36898300 0.11195900 -0.20648200

C 2.39207400 -1.66338500 -0.87817300

C 1.28228300 -1.95330600 -1.85203200

H 0.31361300 -1.95586200 -1.33233700

H 1.22359500 -1.16648800 -2.61063900

H 1.46677400 -2.92371400 -2.31746300

O 0.94780300 -0.45196000 1.83382900

O 3.29824600 -2.43493800 -0.63214200

O -1.64102500 -1.59572100 -0.03857500

O -2.21525400 1.20057200 0.79896400

O 1.38480100 0.55000800 -0.69185500

C 1.67396800 1.97922600 -0.82643400

C 2.83229000 2.18476600 -1.81415600

H 2.97150400 3.25366800 -2.00860700

H 3.78534900 1.80041300 -1.43317200

H 2.61469500 1.68965200 -2.76633000

C 0.37493500 2.54970800 -1.40119800

H 0.15193100 2.08661400 -2.36816100

H -0.46335500 2.35780700 -0.72479400

H 0.47115100 3.63078600 -1.54957700

C 1.98217500 2.60590000 0.53949100

H 2.90208400 2.20146000 0.97945000

H 2.12622900 3.68641300 0.43066400

H 1.15484900 2.43797900 1.23580800

Cu -0.76646700 -0.08308700 1.00177200

**13-trip**

C -3.18390400 -0.82557900 -0.76944000

C -4.12716200 -1.53165500 -1.72068700

H -4.18942100 -2.59122100 -1.44830700

H -5.13034400 -1.09823500 -1.71594200

H -3.71837000 -1.48551800 -2.73644300

C -3.64631600 0.26801900 -0.01702600

H -4.67907300 0.57037200 -0.14233900

C -2.85361700 0.99144100 0.88637800

C -3.45825600 2.15089300 1.65020900

H -2.86848800 3.05600700 1.46678100

H -4.49940200 2.33665500 1.37499800

H -3.40187200 1.94405900 2.72514300

C 2.06653900 -0.34039200 1.29831100

C 3.20593800 -0.53403100 2.26089600

H 3.17091100 -1.53191700 2.72131000

H 4.17945700 -0.41003300 1.77554400

H 3.12486400 0.19195300 3.07959600

C 2.27331900 -0.35334200 -0.19606600

H 3.31868300 -0.18215800 -0.46977500

C 1.85696600 -1.77799000 -0.45823100

C 2.86737700 -2.87793700 -0.63665900

H 3.86921000 -2.48819500 -0.84137700

H 2.91690000 -3.52098700 0.25408700

H 2.56119000 -3.52089300 -1.47083300

O 0.86744200 -0.35716900 1.74001100

O 0.62365800 -2.08498300 -0.33179000

O -2.00151200 -1.30461700 -0.73281200

O -1.62177600 0.76268700 1.14545200

O 1.36516500 0.50231200 -0.89632600

C 1.68559500 1.91412200 -1.05063400

C 2.87231100 2.06439700 -2.01501900

H 3.05201800 3.12297600 -2.23398100

H 3.79888300 1.65881700 -1.59312200

H 2.66538400 1.54640400 -2.95718700

C 0.41346300 2.49648300 -1.67568700

H 0.18120000 1.98462400 -2.61524200

H -0.43333800 2.37013900 -0.99389600

H 0.54138200 3.56483700 -1.88229900

C 1.97425500 2.59510300 0.29514700

H 2.90309600 2.23015100 0.74702600

H 2.08577400 3.67540900 0.14395000

H 1.15466200 2.42429500 0.99895500

Cu -0.55676400 -0.67002000 0.38993500

**14**

C 1.16207600 -1.23611500 -0.32311700

C 2.16262900 -1.98024200 0.53450200

H 1.63325200 -2.48129600 1.35641000

H 2.68021200 -2.73208600 -0.06506800

H 2.87466000 -1.28405400 0.98937500

C 0.55636000 0.05639700 0.30079400

H 0.41132200 -0.10955700 1.37662900

C 1.63168400 1.16460600 0.17664400

C 1.41766700 2.20401900 -0.89745200

H 1.24597400 1.71355000 -1.86255300

H 0.51514800 2.78596400 -0.68473400

H 2.29018500 2.85822500 -0.94589900

O 0.84053300 -1.58888000 -1.43755700

O 2.60988700 1.14251700 0.89944500

O -0.60901600 0.47356500 -0.35854100

C -1.90280500 0.03678900 0.13506900

C -2.20441100 0.71914300 1.47916300

H -3.21956700 0.47582700 1.81182000

H -1.51710300 0.39558000 2.26932800

H -2.12567700 1.80695600 1.37994300

C -2.86999800 0.53062400 -0.94542700

H -2.79504400 1.61741900 -1.05622200

H -2.62602400 0.06961300 -1.90741100

H -3.90343200 0.27805600 -0.68513400

C -1.98126600 -1.49167100 0.25877200

H -1.30442400 -1.87451700 1.03212300

H -2.99642900 -1.79074000 0.54221100

H -1.72590800 -1.96785100 -0.69127800

**15′**

C 3.24826700 -2.58079300 0.36116600

C 4.45871500 -2.89032500 1.21837300

H 4.14154800 -3.03854300 2.25675700

H 4.99705000 -3.77765600 0.87631900

H 5.14380100 -2.03416900 1.20999200

C 2.97640100 -3.37715400 -0.75842700

H 3.64905000 -4.19882100 -0.97396500

C 1.88777300 -3.16776600 -1.62660700

C 1.70779600 -4.08028500 -2.82407500

H 1.70637300 -3.47909500 -3.74026200

H 2.48896700 -4.84147500 -2.89427300

H 0.73007800 -4.57116600 -2.76138900

C 1.06824600 1.50937100 1.69755200

C 0.57616800 2.35663100 2.83266500

H -0.40377100 2.73423800 2.51150900

H 0.43220000 1.74304500 3.72518600

H 1.23790400 3.20214800 3.03384000

C 1.91758200 2.19896400 0.62180000

H 1.44030000 3.16064600 0.40617000

C 3.29646400 2.51310200 1.28936100

C 4.37955300 1.47339900 1.12183300

H 3.97594200 0.46750700 1.28485400

H 4.76248500 1.48931100 0.09580600

H 5.19397200 1.69279200 1.81564100

O 0.81320500 0.31058600 1.64525500

O 3.43565600 3.53225200 1.93474900

O 2.55488000 -1.57603500 0.74636000

O 1.00561100 -2.25814700 -1.50489700

O 2.05871500 1.38417300 -0.51175700

C 2.00538600 2.02281900 -1.83854900

C 3.04654900 3.14745900 -1.93981600

H 3.02482900 3.57298300 -2.94875800

H 2.84907400 3.96640500 -1.23937000

H 4.05885800 2.77028300 -1.75883300

C 2.35893600 0.88237600 -2.79563700

H 3.35731000 0.48913700 -2.57465500

H 1.63500800 0.06732800 -2.70876200

H 2.35118700 1.24593500 -3.82904800

C 0.59000700 2.53936400 -2.11814700

H 0.28726200 3.32435100 -1.41670500

H 0.54805200 2.96625000 -3.12696100

H -0.12701900 1.71890500 -2.04316000

Cu 0.88161300 -0.93280600 -0.03234300

S -1.90357900 0.39387900 0.08113300

C -3.31340600 0.56218500 -1.05737900

C -4.21618400 1.61155600 -0.87898500

C -3.46066200 -0.35035500 -2.10239400

C -5.29246700 1.74016900 -1.75897400

H -4.05762800 2.32180300 -0.07457900

C -4.53929700 -0.21273000 -2.97765100

H -2.71963300 -1.13343600 -2.22559600

C -5.45603100 0.82773700 -2.80442200

H -5.99890900 2.55635100 -1.63210400

H -4.66151900 -0.91544800 -3.79774100

H -6.29461600 0.93163600 -3.48808700

C -2.64918200 -0.55825400 1.46292600

C -2.61297500 -1.95245400 1.40661000

C -3.21830200 0.10128800 2.55359900

C -3.15644800 -2.69567700 2.45617500

H -2.15116900 -2.43841000 0.55183600

C -3.75954900 -0.64829400 3.60006400

H -3.21813900 1.18582200 2.57892100

C -3.73027700 -2.04447500 3.55118300

H -3.12991500 -3.78164800 2.42016300

H -4.20039900 -0.14270300 4.45545700

H -4.15171500 -2.62471900 4.36789900

O -1.71178400 1.77031900 0.66287500

N -0.84785000 -0.39323000 -0.66435000

**15**

C -4.25835900 1.23186000 0.04006700

C -5.05546600 2.51356300 0.15071900

H -4.92882500 3.09989500 -0.76642200

H -6.11978100 2.33162800 0.31798900

H -4.65819300 3.11675900 0.97488800

C -4.91138800 -0.00600500 0.15675700

H -5.98118400 0.00152800 0.32713000

C -4.27244000 -1.25311600 0.06264900

C -5.08392700 -2.52336600 0.19794000

H -4.69568200 -3.11321600 1.03600000

H -6.14659100 -2.32615900 0.35827600

H -4.96119500 -3.13046900 -0.70608600

O -3.00520700 1.39504100 -0.16022200

O -3.02135000 -1.43397800 -0.13523100

Cu -1.72440800 -0.02898800 -0.31101200

S 1.14632500 -0.01238800 0.42632400

C 2.24573900 -1.42096100 0.09973000

C 2.95989400 -1.97206700 1.16481700

C 2.34635400 -1.93937800 -1.19261900

C 3.80127300 -3.05950100 0.92435500

H 2.83574400 -1.56296200 2.16169000

C 3.19069600 -3.02686700 -1.42040400

H 1.75460300 -1.50564300 -1.99161500

C 3.91897200 -3.58333600 -0.36545700

H 4.35901400 -3.50106400 1.74561700

H 3.27464200 -3.44320600 -2.42046800

H 4.57379900 -4.43119500 -0.54769500

C 2.19426700 1.43684400 0.10614800

C 2.28936600 1.95369500 -1.18735300

C 2.87403000 2.01977600 1.17669300

C 3.09325200 3.07226500 -1.41060300

H 1.72440300 1.49429100 -1.99140800

C 3.67478600 3.13854900 0.94078400

H 2.75476900 1.60996100 2.17387300

C 3.78691400 3.66122600 -0.34992400

H 3.17251400 3.48729000 -2.41159100

H 4.20528500 3.60503200 1.76632000

H 4.41021700 4.53326600 -0.52856400

O 0.89284200 -0.02001500 1.89263300

N 0.05700300 -0.03062000 -0.66328500

**16′**

C -0.63442300 2.65160100 -1.39097900

C -0.22994200 3.57839000 -2.52344300

H -0.56147700 3.15118800 -3.47667500

H -0.64569900 4.58376600 -2.41693300

H 0.86347100 3.65088200 -2.56884800

C -1.40667400 3.16782200 -0.33439000

H -1.67785600 4.21639200 -0.37851200

C -1.83171300 2.43558800 0.78986100

C -2.60261600 3.15289000 1.88354100

H -2.06312200 3.05512900 2.83280000

H -2.75518900 4.21364100 1.66775000

H -3.57658500 2.66867900 2.01784100

C 1.88535300 -1.53430900 -1.21272900

C 2.58881000 -2.59331500 -2.02301300

H 2.64610000 -3.51153200 -1.42222300

H 2.02437500 -2.80540900 -2.93339200

H 3.61451900 -2.29164600 -2.25516400

C 2.73019800 -0.80997500 -0.14946000

H 3.38714700 -1.55947200 0.30548200

C 3.66931700 0.17604200 -0.91820700

C 3.22697600 1.61456200 -0.98589300

H 2.19265100 1.66467400 -1.34974500

H 3.22139200 2.06345300 0.01268200

H 3.90383400 2.16695000 -1.64086800

O 0.71121100 -1.25819600 -1.40375400

O 4.66892100 -0.25442200 -1.45867700

O -0.21447000 1.45092600 -1.48725800

O -1.63576900 1.19238900 0.99685500

O 1.91632500 -0.15058700 0.78240700

C 2.20106900 -0.33168600 2.20763600

C 3.66248400 0.01777800 2.52919600

H 3.82329900 -0.03526200 3.61129900

H 4.37558000 -0.67054400 2.06234400

H 3.90008700 1.03622800 2.20428700

C 1.25867500 0.66168600 2.89112000

H 1.51108400 1.68728600 2.60181100

H 0.22040600 0.47748200 2.60302700

H 1.34739300 0.57702300 3.97972800

C 1.86710000 -1.77114700 2.62267700

H 2.51067400 -2.50348700 2.12068100

H 2.01299100 -1.89806000 3.70103400

H 0.82477000 -2.00400400 2.38517900

Cu -0.82048200 -0.07833300 -0.32172700

C -2.75510600 -1.65184800 1.33555100

H -2.97901300 -2.65146200 1.75472400

H -2.39362500 -1.03991200 2.17018500

C -1.67732400 -1.79200200 0.25705700

H -0.80752600 -2.32769200 0.66897100

C -2.21253400 -2.55355000 -0.96613600

H -1.45507800 -2.61051600 -1.75720700

H -2.42980500 -3.59959400 -0.67531800

C -3.51114500 -1.94159900 -1.51832300

H -3.30032800 -0.93682500 -1.91630300

H -3.88308600 -2.54052900 -2.36072400

C -4.06709000 -1.05862600 0.79461100

H -4.82877300 -1.04739300 1.58636900

H -3.89096600 -0.01367800 0.50866400

C -4.58494800 -1.83771200 -0.42458700

H -5.49316000 -1.36543600 -0.82296900

H -4.87304600 -2.85307700 -0.11101500

**16**

C -2.56297200 1.21188700 0.22459700

C -3.48481900 2.37853700 0.50825100

H -3.48216600 3.06069100 -0.34963200

H -4.51102200 2.06525900 0.71505000

H -3.10150900 2.94241100 1.36660000

C -3.07369900 -0.09752500 0.26414700

H -4.12646700 -0.21060200 0.49446300

C -2.33982900 -1.27602400 0.03336500

C -3.05210500 -2.61243500 0.10896600

H -2.55659900 -3.24617000 0.85291600

H -4.10907400 -2.51232700 0.36746900

H -2.96508100 -3.12321300 -0.85687400

O -1.35019700 1.52717500 -0.03571300

O -1.09364200 -1.35578600 -0.24511300

Cu 0.03829500 0.24340200 -0.42759300

C 1.74327500 -0.56808800 -0.91735300

H 1.48208700 -1.13579900 -1.82126700

C 2.24725400 -1.50775000 0.17969500

C 2.75809700 0.53873200 -1.23790300

H 3.10884200 -2.08309600 -0.20611200

H 1.47585700 -2.24431100 0.43468500

C 2.70025900 -0.74692000 1.43802000

H 2.36282600 1.23897400 -1.98717200

H 3.65065100 0.07850700 -1.70176600

C 3.21030000 1.30271100 0.01961000

H 3.11644300 -1.44677900 2.17440400

H 1.82385000 -0.27939900 1.91442700

C 3.73177200 0.34196600 1.09976500

H 2.36216900 1.87686000 0.42610000

H 3.98320800 2.03695000 -0.24191400

H 4.00860600 0.89986900 2.00375300

H 4.65235800 -0.13812400 0.73472300

**17′**

C 0.79834900 -3.06492500 -0.54502900

C 0.21428600 -4.34871800 -1.09858300

H 0.17645000 -4.28788700 -2.19202100

H 0.78937300 -5.23105800 -0.80709100

H -0.81795800 -4.46663600 -0.74787300

C 1.92036100 -3.12690400 0.29485700

H 2.32990000 -4.10382600 0.52243500

C 2.53658300 -2.00494100 0.87590100

C 3.71462000 -2.20214900 1.80826700

H 3.47142200 -1.78522000 2.79236200

H 3.98876400 -3.25382300 1.92284400

H 4.57651200 -1.64302200 1.42734400

C -2.02907300 0.67749200 -1.53015000

C -2.95847600 1.26622400 -2.55754000

H -3.01391000 2.35085100 -2.39149100

H -2.57006100 1.08463400 -3.56169700

H -3.97010200 0.86551200 -2.44412500

C -2.59353400 0.48578300 -0.10904600

H -3.22183800 1.35615400 0.10703500

C -3.56570200 -0.73605000 -0.15366900

C -3.06089700 -2.03136600 0.42840400

H -2.09971300 -2.28720200 -0.03329800

H -2.87005000 -1.92311400 1.50085600

H -3.80180600 -2.81426900 0.25448600

O -0.88576400 0.35667000 -1.81372300

O -4.65608500 -0.61094000 -0.67543000

O 0.19020000 -1.99534900 -0.89584300

O 2.19353700 -0.78952400 0.69248400

O -1.56871400 0.30641900 0.83277000

C -1.53191000 1.21993800 1.98734400

C -2.81939500 1.07531800 2.81196900

H -2.75894000 1.70778700 3.70425700

H -3.71178000 1.38643200 2.25714600

H -2.95918400 0.03980200 3.14145600

C -0.32818700 0.73164300 2.79550100

H -0.45525200 -0.31939600 3.07667400

H 0.59262600 0.83113200 2.21588700

H -0.22995400 1.32459800 3.71141000

C -1.30987800 2.66085000 1.51163200

H -2.17958900 3.05739900 0.97282000

H -1.14622500 3.31432600 2.37621600

H -0.43252800 2.69763400 0.85787400

Cu 0.76647300 -0.15964700 -0.50886600

O 1.14822300 1.65453500 -0.54059000

C 2.41805900 2.25787600 -0.67024500

C 2.17047000 3.65185300 -1.28504500

H 3.10772000 4.20929200 -1.40940200

H 1.69123700 3.55143900 -2.26522000

H 1.50560600 4.23632300 -0.63888200

C 3.08785500 2.41622400 0.71087500

H 2.44434400 3.00880600 1.37224200

H 3.24192900 1.43542200 1.16939400

H 4.05829000 2.92461800 0.63599500

C 3.33355900 1.44842200 -1.61179700

H 3.53422000 0.45581600 -1.19287800

H 2.85123000 1.32401000 -2.58902500

H 4.29783300 1.94932100 -1.76782700

**17**

C 2.40636700 1.24555400 0.06135500

C 3.20798400 2.52211800 0.18641400

H 2.80593000 3.12413000 1.00911300

H 4.26958000 2.33391800 0.36282700

H 3.09330100 3.11308000 -0.72935100

C 3.05287000 0.00334100 0.16707800

H 4.12245200 0.00569200 0.33855200

C 2.41175900 -1.24168000 0.06200500

C 3.21876400 -2.51474000 0.18784900

H 3.10546900 -3.10742600 -0.72696700

H 4.27976300 -2.32191500 0.36285400

H 2.82014800 -3.11729900 1.01183800

O 1.15294700 1.41636800 -0.13993600

O 1.15904500 -1.41788600 -0.13918200

Cu -0.11441700 -0.00379300 -0.34351800

O -1.85878700 -0.00389300 -0.72038700

C -2.99170900 -0.00011100 0.13173500

C -3.80117800 1.26829900 -0.19716200

H -4.71784600 1.31805900 0.40268700

H -3.20299700 2.16315700 0.00774400

H -4.07524500 1.27543800 -1.25696700

C -3.81238900 -1.26012200 -0.20156300

H -4.08622600 -1.26123200 -1.26145100

H -3.22222000 -2.16092800 0.00043900

H -4.72962900 -1.30382700 0.39788100

C -2.58258800 -0.00447300 1.61411700

H -1.98967600 -0.89851300 1.84496700

H -1.98155800 0.88335800 1.84799800

H -3.45806500 -0.00153700 2.27493000

**18-trip**

C 4.45495100 -0.20434200 -0.34112900

C 5.79736500 0.43679700 -0.62378000

H 6.00885100 1.19147600 0.14221800

H 6.61320200 -0.28990800 -0.64779200

H 5.75315400 0.95944000 -1.58625400

C 4.37244200 -1.59575100 -0.17941100

H 5.28670000 -2.17073000 -0.26571800

C 3.18189700 -2.29216500 0.08930000

C 3.21836700 -3.79824100 0.24520500

H 2.55679500 -4.25461300 -0.49985400

H 4.22513600 -4.20775800 0.13127300

H 2.82697200 -4.07053800 1.23196700

O 3.47245700 0.61204000 -0.27422100

O 2.02608800 -1.76534000 0.22301400

Cu 1.61524000 0.12515200 0.03211400

S -1.32371800 -0.26606300 -0.48507600

C -2.01969900 -1.88778000 -0.03680100

C -2.53606500 -2.68148500 -1.06182500

C -2.01260700 -2.31845300 1.29125600

C -3.07195300 -3.93028100 -0.74296400

H -2.49799900 -2.32673100 -2.08615800

C -2.55070800 -3.56944500 1.59597800

H -1.57207900 -1.69187100 2.05904200

C -3.08237600 -4.37144400 0.58253500

H -3.47550600 -4.55991600 -1.53110100

H -2.54819900 -3.92004200 2.62432800

H -3.49903900 -5.34516800 0.82557700

C -2.72622600 0.86558900 -0.27183100

C -3.11195000 1.26517600 1.00947200

C -3.39357900 1.31642000 -1.41022800

C -4.19875700 2.12834300 1.14879200

H -2.55733500 0.91923000 1.87531800

C -4.47610400 2.18567700 -1.25779500

H -3.05215400 0.99971400 -2.38970200

C -4.88002700 2.58695700 0.01713000

H -4.50897800 2.44856000 2.13953100

H -5.00044700 2.55086600 -2.13645000

H -5.72366900 3.26227800 0.13063200

O -1.04234200 -0.32069100 -1.94252500

N -0.26591000 0.04735000 0.61745300

O 0.85860900 1.88232700 -0.36758900

C 1.05440300 3.04296300 0.42122000

C 2.22816200 3.80680700 -0.23801000

H 2.42848200 4.73715200 0.30817400

H 3.12832300 3.18680100 -0.23590700

H 1.97996200 4.05644900 -1.27464200

C -0.22888300 3.88828800 0.35577300

H -1.06294100 3.34988400 0.81606900

H -0.09793200 4.84303300 0.87971100

H -0.49013700 4.09592500 -0.68701600

C 1.39852700 2.69806500 1.88032200

H 1.53295800 3.60913100 2.47618900

H 0.59335700 2.10503800 2.32589200

H 2.32985800 2.12159600 1.93371700

**18-sing**

C -4.18051800 0.69788800 -0.60992800

C -5.54307800 0.13411900 -0.95323800

H -5.99797600 -0.29074500 -0.05118500

H -6.21224900 0.89057800 -1.37035700

H -5.42612600 -0.68435300 -1.67191000

C -3.91878700 2.06060500 -0.80020900

H -4.69807700 2.68802000 -1.21428900

C -2.70805900 2.65473500 -0.41975300

C -2.50677300 4.14517600 -0.58349500

H -1.63175400 4.31758000 -1.21945100

H -3.37985100 4.63654000 -1.01984400

H -2.28912900 4.59119700 0.39328100

O -3.35128600 -0.15695600 -0.14544700

O -1.70805000 2.04373400 0.09055200

Cu -1.47731500 0.14869500 0.27694100

S 1.25844200 0.59986100 -0.42059900

C 2.75956300 1.24148500 0.36527800

C 3.60847600 2.01126400 -0.43126300

C 3.03953200 0.99661400 1.71135200

C 4.77618200 2.52912700 0.13158500

H 3.34389300 2.20730100 -1.46489400

C 4.20879700 1.52381300 2.26145300

H 2.34042100 0.42584200 2.31170200

C 5.07724500 2.28365800 1.47324700

H 5.44588500 3.13008000 -0.47726000

H 4.43746800 1.34512300 3.30845600

H 5.98609000 2.69111900 1.90768700

C 1.81574900 -0.98341800 -1.15160500

C 2.42454800 -1.95053000 -0.35037800

C 1.59537300 -1.20523000 -2.51069300

C 2.82762000 -3.15581700 -0.92429200

H 2.58481600 -1.76467800 0.70618200

C 1.98807100 -2.42210300 -3.07334600

H 1.13047700 -0.42851600 -3.10750600

C 2.60535100 -3.39377200 -2.28397600

H 3.31133200 -3.91032900 -0.30993900

H 1.81700400 -2.60550000 -4.13060000

H 2.91639100 -4.33616900 -2.72671300

O 0.96565100 1.44809300 -1.61035000

N 0.29651200 0.45553900 0.79034100

O -1.23282400 -1.65352100 0.32393100

C -1.38278500 -2.35936300 1.54798100

C -1.93657300 -3.73299800 1.10137700

H -2.04291800 -4.39779000 1.96753300

H -2.91215100 -3.61096100 0.62165100

H -1.25281700 -4.19844900 0.38463900

C -0.03113500 -2.54504100 2.25786500

H 0.39686000 -1.57135900 2.51194100

H -0.14813400 -3.13201400 3.17749100

H 0.66905100 -3.06889000 1.59988900

C -2.39066100 -1.68484100 2.49894800

H -2.55196000 -2.30300300 3.39021600

H -2.01111300 -0.71199200 2.83921600

H -3.35068900 -1.53034200 1.99906200

**19-trip**

C -3.24332000 -2.47059900 -0.54572100

C -3.79899900 -3.82440400 -0.15748900

H -3.90043100 -3.87694800 0.93242300

H -4.76794600 -4.02867300 -0.61954600

H -3.08783500 -4.60450100 -0.45180600

C -3.96811700 -1.64968600 -1.42681300

H -4.91273600 -2.02420400 -1.80229900

C -3.54862600 -0.38166900 -1.86319700

C -4.41951300 0.40512700 -2.81926600

H -3.83749100 0.66292900 -3.71094400

H -5.31522600 -0.14443600 -3.11891400

H -4.71799700 1.34780000 -2.34603300

O -2.11614700 -2.18117800 -0.01728700

O -2.46459000 0.20291200 -1.51849600

Cu -1.13432900 -0.55251500 -0.34742900

S 1.79893100 -0.40006800 -0.61756000

C 2.69071400 1.14430700 -0.96783100

C 3.52048900 1.19686800 -2.08889900

C 2.52289100 2.25036500 -0.13261800

C 4.20398400 2.38099100 -2.37040500

H 3.60597500 0.32818700 -2.73275600

C 3.21150600 3.42862100 -0.42406600

H 1.84838900 2.18557100 0.71434300

C 4.05258800 3.49283700 -1.53827500

H 4.84902700 2.43634500 -3.24299500

H 3.08623900 4.29802900 0.21547500

H 4.58527700 4.41320100 -1.76209600

C 2.90716900 -1.28587900 0.51940100

C 2.82704100 -1.04983600 1.89300900

C 3.81346300 -2.20597700 -0.00991000

C 3.68278300 -1.74291300 2.74997900

H 2.09153600 -0.34987500 2.27471100

C 4.66420900 -2.89384600 0.85720100

H 3.82939100 -2.38674900 -1.07928100

C 4.60184500 -2.66016000 2.23308700

H 3.62768900 -1.57092400 3.82142200

H 5.37067600 -3.61638000 0.45792900

H 5.26526500 -3.19864500 2.90450100

O 1.81417000 -1.17670700 -1.88782500

N 0.53333900 -0.02400500 0.17709100

C -2.41916500 0.54655900 2.16367200

H -2.22213800 -0.50580400 2.35966500

C -3.82984800 0.96521900 1.87398800

C -1.44995800 1.56202200 2.68915800

H -4.39028500 1.04161100 2.82821400

H -4.34946600 0.19794600 1.28584700

C -3.90243900 2.33281000 1.16455000

H -0.42511100 1.18087000 2.61252700

H -1.64102900 1.72449200 3.76964600

C -1.57284500 2.91941200 1.96805700

H -4.94584800 2.66989400 1.11059000

H -3.54521700 2.21535600 0.13290600

C -3.03520800 3.38122000 1.87741900

H -1.16311800 2.81912500 0.95380800

H -0.96520800 3.67297300 2.48612400

H -3.09600000 4.34247600 1.35071800

H -3.42842900 3.55385400 2.89126000

**19-sing**

C 4.03028700 1.47474700 -0.04643400

C 5.54173000 1.38909700 -0.04596500

H 5.88030700 0.90886000 -0.97129900

H 6.01436800 2.37039300 0.04212600

H 5.87125300 0.75497400 0.78507700

C 3.40633500 2.71715900 0.05334700

H 4.03377100 3.59619100 0.13920400

C 2.00546200 2.89443600 0.05053300

C 1.42725900 4.28736400 0.19169500

H 0.87802200 4.35124700 1.13840900

H 2.19369000 5.06651300 0.16996000

H 0.69945300 4.46076000 -0.60768400

O 3.44378600 0.33141400 -0.14223400

O 1.15880000 1.95678000 -0.05826700

Cu 1.55575400 0.00250500 -0.16670800

S -1.28982100 -0.01467500 0.49799400

C -1.89646700 1.66826200 0.08552700

C -2.36296000 2.50278300 1.10058600

C -1.89229500 2.08324600 -1.24631000

C -2.83695400 3.77547700 0.77208100

H -2.33609300 2.15670500 2.12833100

C -2.37261400 3.35316700 -1.56745500

H -1.49596700 1.42455400 -2.01317800

C -2.84549700 4.19897000 -0.55900600

H -3.19923200 4.43541500 1.55615900

H -2.37410600 3.68499300 -2.60251200

H -3.21883100 5.18803900 -0.81155400

C -2.76304700 -1.00239600 0.09662100

C -2.90656100 -1.59219800 -1.16033200

C -3.73235100 -1.16180100 1.08775200

C -4.04915400 -2.34736600 -1.42839800

H -2.12228800 -1.47026900 -1.89932500

C -4.87073900 -1.92090400 0.80929900

H -3.57923600 -0.71224700 2.06321500

C -5.03111300 -2.50954400 -0.44707900

H -4.17060900 -2.81278900 -2.40292900

H -5.62866100 -2.05644000 1.57613300

H -5.91849700 -3.09959200 -0.66029000

O -1.16695400 -0.01057500 1.98549900

N -0.20762400 -0.55411200 -0.45056000

C 1.61983400 -2.71000900 -1.29062100

H 0.53118100 -2.69598000 -1.40109700

H 2.05407600 -2.24094300 -2.18214200

C 2.00837000 -1.96190200 -0.03632100

H 3.08291000 -1.76866600 0.01579900

C 1.47144400 -2.57060500 1.24089500

H 1.78639200 -1.99739800 2.12042100

H 0.37797100 -2.57368100 1.22421100

C 1.98605100 -4.03084200 1.34946300

H 1.57181200 -4.48891800 2.25664600

H 3.07939800 -4.02927000 1.46656700

C 1.60467600 -4.84525000 0.10504800

H 0.51010800 -4.93590300 0.05415700

H 2.00242300 -5.86562900 0.18334700

C 2.11771100 -4.17620300 -1.17813100

H 3.21712800 -4.19069800 -1.18279800

H 1.79139200 -4.73435200 -2.06516000

**20-MECP**

C -3.73230600 -0.26825800 -1.90821700

C -4.52733400 -1.11272200 -2.88486200

H -4.81778100 -2.05483600 -2.40615200

H -5.42173000 -0.60191400 -3.24938300

H -3.89033400 -1.36993000 -3.73935200

C -4.15133400 1.04183800 -1.63339600

H -5.05247000 1.38960000 -2.12467500

C -3.48080600 1.95533500 -0.78944500

C -4.05377100 3.35027700 -0.62688100

H -3.31764700 4.08544700 -0.97140500

H -4.98493500 3.48855100 -1.18223700

H -4.23490800 3.54610500 0.43598500

O -2.70948400 -0.84970400 -1.40429500

O -2.41715300 1.71665700 -0.13737700

Cu -1.43014400 -0.05693500 -0.15496800

S 1.35843300 0.29332900 -0.60892200

C 2.43198300 1.51904600 0.21248500

C 3.08472100 2.47210800 -0.57237700

C 2.58318700 1.49788000 1.59886500

C 3.91025400 3.41266300 0.04518900

H 2.92564400 2.47975400 -1.64514000

C 3.40891500 2.44505200 2.20784500

H 2.04588300 0.75905300 2.18455200

C 4.07409900 3.39828800 1.43277500

H 4.42077800 4.15936300 -0.55689200

H 3.52966900 2.44109500 3.28745200

H 4.71717500 4.13281600 1.91038100

C 2.54108300 -1.02578400 -1.03589300

C 2.82882600 -2.03836400 -0.11919200

C 3.11683900 -1.01305200 -2.30692500

C 3.71718900 -3.05106400 -0.48323200

H 2.34590800 -2.03732700 0.85254200

C 4.00408500 -2.03116800 -2.66147600

H 2.84795300 -0.22724500 -3.00443200

C 4.30565100 -3.04644600 -1.75130800

H 3.94388100 -3.84825500 0.21932700

H 4.45259600 -2.03355900 -3.65133400

H 4.99589200 -3.83775700 -2.03120100

O 0.98127400 0.92424300 -1.90922400

N 0.34881200 -0.28495100 0.39250500

C -2.48391300 -0.93880500 1.54833600

H -3.40577500 -0.65784300 1.03718100

C -2.18882700 -0.14025000 2.78932100

C -2.20364100 -2.41692800 1.57469000

H -3.05827800 -0.29954300 3.45922600

H -2.18750600 0.93249100 2.56508400

C -0.90908900 -0.56582200 3.52657100

H -2.22602800 -2.83144900 0.56013700

H -3.06330900 -2.88064600 2.09959900

C -0.90954900 -2.79109600 2.31373100

H -0.87774100 -0.07680800 4.50850300

H -0.04672400 -0.21209200 2.95108900

C -0.82242400 -2.09293600 3.68080900

H -0.05813800 -2.48596000 1.69517500

H -0.86175900 -3.88057000 2.43335600

H 0.11446000 -2.36574800 4.18329500

H -1.63733600 -2.44858700 4.33131000

**21-ts**

C -4.33736100 0.06714100 0.31534400

C -5.59798400 0.79261700 0.74687200

H -5.77485800 1.64556600 0.08091600

H -6.47986000 0.14666700 0.74014400

H -5.45859300 1.19727000 1.75626600

C -4.41565700 -1.28450400 -0.04043500

H -5.39008900 -1.75684700 0.01240800

C -3.32862600 -2.08008700 -0.47387500

C -3.59280700 -3.52980600 -0.84850700

H -2.91567100 -4.17791700 -0.28127700

H -4.62607600 -3.83753200 -0.66538900

H -3.36256700 -3.67615500 -1.91073000

O -3.27731500 0.78915000 0.31378300

O -2.12828000 -1.69161800 -0.59173900

Cu -1.46961100 0.19580700 -0.11707400

S 1.33110200 -0.25894300 0.54792500

C 1.29717700 -2.06795400 0.30690400

C 1.59886600 -2.89517400 1.38991000

C 0.97774300 -2.59168500 -0.94566200

C 1.58915000 -4.27898200 1.20607600

H 1.81656700 -2.45575300 2.35737500

C 0.97660700 -3.97588400 -1.11858800

H 0.70693500 -1.92562700 -1.75778500

C 1.28437700 -4.81747400 -0.04654800

H 1.81559000 -4.93511400 2.04216200

H 0.72408800 -4.39669100 -2.08788100

H 1.27936900 -5.89532000 -0.18565500

C 3.00504600 0.16426900 -0.01490900

C 3.22654100 0.54096800 -1.34109100

C 4.05098700 0.10702900 0.90821200

C 4.52304000 0.86066500 -1.74720900

H 2.38870500 0.59811800 -2.02760400

C 5.34307100 0.43114800 0.49104700

H 3.84500600 -0.16600500 1.93773800

C 5.57966600 0.80344800 -0.83440900

H 4.70693800 1.15790000 -2.77606000

H 6.16308300 0.39770900 1.20307600

H 6.58707800 1.05441200 -1.15525400

O 1.31834900 -0.05746800 2.02636400

N 0.36619200 0.45920800 -0.43163300

C -0.96536000 2.83674100 -1.15398900

H -0.11139200 2.72741400 -1.83248000

H -1.82614300 2.34303800 -1.61642000

C -0.61574900 2.21832500 0.18148500

H -1.47261100 2.15321400 0.86020200

C 0.55779700 2.88064000 0.86696300

H 0.78898800 2.38442300 1.81196800

H 1.44611400 2.81931300 0.22842500

C 0.20524300 4.37056600 1.10199900

H 1.05597300 4.86299400 1.58939800

H -0.64222200 4.44566300 1.79828800

C -0.14806200 5.07112400 -0.21761300

H -0.42975200 6.11555500 -0.03237700

H 0.74182700 5.09456700 -0.86341700

C -1.28494900 4.34004000 -0.94501300

H -2.21225300 4.43099800 -0.36284800

H -1.48101400 4.79883600 -1.92244500

**22**

C 4.05208200 -1.51068900 -0.08109000

C 4.79581400 -2.83897100 -0.13649100

H 4.45933500 -3.40730800 -1.01165800

H 5.88228100 -2.72209100 -0.18375200

H 4.54087300 -3.43386700 0.74879700

C 4.81636700 -0.31963300 -0.06952200

H 5.89468300 -0.43125400 -0.10441100

C 4.32221600 0.99423800 -0.01364700

C 5.31639000 2.14432600 -0.00570600

H 5.17611200 2.73941600 0.90447400

H 6.35599600 1.80909800 -0.05515200

H 5.11377100 2.80703400 -0.85541900

O 2.78742500 -1.58837400 -0.04772800

O 3.09627400 1.35633100 0.03350300

Cu 1.60045600 0.14461600 0.07189200

S -1.29267400 0.58263200 0.77341500

C -0.76654700 2.19163700 0.16216400

C -0.83808700 3.24942600 1.06802300

C -0.35853600 2.38152100 -1.16178300

C -0.47678900 4.52850900 0.63853900

H -1.15100000 3.06184600 2.08916300

C -0.00754500 3.66436900 -1.57570300

H -0.29078600 1.54193200 -1.84366700

C -0.06517600 4.73561800 -0.67797300

H -0.51433200 5.35891800 1.33749200

H 0.32377300 3.82483200 -2.59735200

H 0.21646300 5.73176200 -1.00739300

C -2.96064200 0.38677200 0.06459500

C -3.11784200 0.08773900 -1.29117200

C -4.06184700 0.56879200 0.90245700

C -4.40600000 -0.03155200 -1.81289700

H -2.24978200 -0.06653300 -1.92306000

C -5.34602500 0.44484800 0.36853400

H -3.90339300 0.79121100 1.95186500

C -5.51796000 0.14732600 -0.98497200

H -4.54042800 -0.26824000 -2.86447200

H -6.21065600 0.57978500 1.01209900

H -6.51902900 0.05198900 -1.39612100

O -1.49504000 0.68476800 2.24100000

N -0.27710600 -0.42926800 0.11795500

C -0.30442300 -2.56443500 -1.12710400

H -1.17984800 -2.27258300 -1.72604200

H 0.58703400 -2.17469100 -1.62906500

C -0.41220200 -1.92469700 0.26592100

H 0.47733300 -2.22607800 0.83001600

C -1.63593200 -2.46773900 1.02610300

H -1.70011100 -2.01409600 2.02176100

H -2.56149200 -2.21910800 0.49220300

C -1.53465500 -3.99931600 1.14848700

H -2.41118200 -4.38285700 1.68557600

H -0.65648500 -4.25594800 1.75829500

C -1.41308500 -4.66956300 -0.22814100

H -1.30454300 -5.75562500 -0.11270900

H -2.34441600 -4.50584700 -0.79107800

C -0.23054300 -4.09642400 -1.02388700

H 0.71171000 -4.37100200 -0.52947500

H -0.19605700 -4.53619600 -2.02871000

**23-MECP**

C 4.20323900 -1.54772200 0.32895600

C 4.89413000 -2.81768900 0.78587700

H 4.55427600 -3.65679000 0.16756500

H 5.98344900 -2.74935400 0.73123200

H 4.59937700 -3.04020100 1.81811700

C 4.97830700 -0.44975300 -0.08174300

H 6.05602900 -0.55940900 -0.05109200

C 4.46330500 0.78145800 -0.52663400

C 5.42905700 1.88089400 -0.92614500

H 5.25110100 2.76628200 -0.30514600

H 6.47409700 1.57604000 -0.82515600

H 5.23823300 2.17586900 -1.96438000

O 2.92730100 -1.58737200 0.35989000

O 3.22611300 1.08103400 -0.63318600

Cu 1.75587400 -0.11443300 -0.17745300

S -1.03800000 0.69665900 0.58419500

C -0.79644600 2.48656000 0.33236800

C -0.96689000 3.33983300 1.42289800

C -0.43198400 2.97291900 -0.92538900

C -0.77308800 4.71142800 1.24489300

H -1.22838200 2.92375300 2.39005600

C -0.24467900 4.34544100 -1.09086100

H -0.27935100 2.28450500 -1.75037700

C -0.41685500 5.21321700 -0.00862600

H -0.89459700 5.38574400 2.08821900

H 0.04457200 4.73584100 -2.06252300

H -0.26564600 6.28111600 -0.14146600

C -2.71871000 0.44997600 -0.05860600

C -2.89482300 0.13651400 -1.40773600

C -3.80971800 0.59819400 0.80005000

C -4.18929700 -0.01861600 -1.90609000

H -2.02304600 -0.00210700 -2.03890700

C -5.10030900 0.43876000 0.29125200

H -3.64233000 0.81827800 1.84911900

C -5.29027300 0.13753700 -1.05956000

H -4.33716500 -0.26679500 -2.95346000

H -5.95664300 0.54632200 0.95136000

H -6.29665600 0.01864200 -1.45174500

O -1.13558800 0.50220500 2.05929500

N -0.07200600 -0.07254300 -0.33664300

C -0.46226000 -2.62430700 0.88233800

H 0.23322700 -2.07039100 1.50372400

C 0.08251200 -3.40675900 -0.26075300

C -1.80130400 -2.95202500 1.45279900

H 0.53230800 -4.33736900 0.14610100

H 0.91281100 -2.86560900 -0.72611800

C -0.99239100 -3.79785700 -1.29034000

H -2.17487000 -2.10846600 2.04541300

H -1.65818200 -3.77992400 2.17996100

C -2.83390000 -3.40757000 0.40227900

H -0.57388900 -4.50368300 -2.01880100

H -1.28891900 -2.89937600 -1.84725100

C -2.22617700 -4.39841200 -0.60173600

H -3.20592600 -2.53050500 -0.14203800

H -3.69846500 -3.85595200 0.90770800

H -2.97734900 -4.68167200 -1.34982700

H -1.93763300 -5.32402800 -0.07968700

**24**

C -4.03046100 -1.80539500 0.24928100

C -4.78013300 -3.11116100 0.48420600

H -4.44555400 -3.55614300 1.42877000

H -5.86620500 -2.98482600 0.51493500

H -4.52697200 -3.82179000 -0.31158700

C -4.79233600 -0.62116800 0.08517200

H -5.87069200 -0.72449100 0.13972700

C -4.29856500 0.67191000 -0.14614000

C -5.29169800 1.81222900 -0.29954500

H -5.15776400 2.28157200 -1.28143300

H -6.33101400 1.48779700 -0.19884200

H -5.08274800 2.58171100 0.45314400

O -2.76820800 -1.89064400 0.21921200

O -3.07221000 1.02799000 -0.24986400

Cu -1.59209700 -0.17298800 -0.12254100

S 1.43462100 -0.16013300 -0.74016700

C 1.47741900 1.48869000 -0.03531000

C 1.94811800 2.51410700 -0.85651800

C 1.08029300 1.72134300 1.28442400

C 2.01840700 3.80874300 -0.33973600

H 2.22572500 2.29881900 -1.88247300

C 1.15692600 3.02011500 1.78474700

H 0.69411500 0.91036600 1.89067600

C 1.62566800 4.06001600 0.97607400

H 2.37167000 4.61966200 -0.96969200

H 0.83963800 3.22074900 2.80370500

H 1.67805200 5.07009500 1.37245400

C 2.92265600 -1.01862100 -0.17184900

C 3.98635600 -1.15840100 -1.06555700

C 2.98237500 -1.53363300 1.12654700

C 5.13599200 -1.82826200 -0.64452700

H 3.89708500 -0.76325200 -2.07151800

C 4.13792800 -2.20069100 1.53327000

H 2.13580200 -1.42699000 1.79667100

C 5.21221900 -2.34531900 0.65089800

H 5.96885200 -1.94989100 -1.33091300

H 4.19652200 -2.61051600 2.53734500

H 6.10904000 -2.86687400 0.97313900

O 1.55514300 -0.04324100 -2.21111900

N 0.20945300 -0.84658900 -0.01224800

H 0.25037200 -1.86094600 -0.14049200

**25-ts**

S 1.51182700 0.08819300 -0.60787400

C 1.90151700 1.70663900 0.10560300

C 2.47840200 2.67428000 -0.71784900

C 1.62733900 1.96176200 1.44969800

C 2.79470300 3.92170100 -0.17749600

H 2.65182000 2.45260600 -1.76543500

C 1.94788600 3.21241700 1.97919600

H 1.15063900 1.19736700 2.05375400

C 2.53352000 4.18900900 1.16867400

H 3.23977600 4.68541500 -0.80929400

H 1.73610600 3.42539800 3.02343700

H 2.78178100 5.16131500 1.58577900

C 3.00806600 -0.89060400 -0.29584500

C 3.17920300 -1.53469800 0.93136100

C 3.96163900 -0.99023200 -1.30995700

C 4.33526600 -2.28591500 1.14568900

H 2.40888400 -1.45916800 1.69142500

C 5.11385100 -1.74548300 -1.08414900

H 3.78394700 -0.49999300 -2.26114400

C 5.30194900 -2.38851300 0.14144000

H 4.47845600 -2.79527600 2.09462900

H 5.86025600 -1.83626000 -1.86836300

H 6.19965500 -2.97637200 0.31254700

O 1.44255000 0.28162900 -2.07998600

N 0.38035400 -0.53687500 0.23947200

H -0.12941400 -1.63851100 -0.07083400

O -1.24172800 -2.13086800 -0.39381100

C -1.78678600 -3.24648600 0.32231500

C -1.99479800 -2.88767100 1.80450600

H -2.37494700 -3.74662200 2.37118000

H -1.04918900 -2.57132800 2.26083900

H -2.71845900 -2.07028800 1.90213000

C -3.12211900 -3.62063700 -0.33581300

H -2.96152600 -3.87939400 -1.38807900

H -3.58366400 -4.48029200 0.16637900

H -3.81182300 -2.77313000 -0.29204800

C -0.77975600 -4.40138300 0.18924300

H -1.15231200 -5.30991500 0.67772800

H -0.59973800 -4.62417100 -0.86768500

H 0.17877500 -4.13408200 0.64993300

C -2.53542500 2.52673000 -0.23305700

C -2.20259800 4.00048800 -0.32721300

H -1.64576300 4.18449700 -1.25336000

H -3.09308700 4.63382900 -0.31290000

H -1.54329900 4.27778900 0.50255400

C -3.87857100 2.11619300 -0.21653200

H -4.64329700 2.88216200 -0.26295800

C -4.29373600 0.77753400 -0.14491500

C -5.77245900 0.45298100 -0.12816200

H -6.00800200 -0.12382500 0.77343400

H -6.39898500 1.34783100 -0.15674800

H -6.01316600 -0.18262800 -0.98801800

O -1.52167700 1.75080600 -0.17534800

O -3.51966900 -0.23981300 -0.09326100

Cu -1.57839700 -0.18560800 -0.11656500

**26-trip**

C 0.46550600 -4.42260400 -0.45714000

C 1.39834100 -5.37065600 -1.18635000

H 2.37660100 -5.36713800 -0.69181800

H 1.01756800 -6.39466000 -1.21867400

H 1.55346800 -5.01236100 -2.21039900

C -0.71724400 -4.92645500 0.11124000

H -0.92159300 -5.98455700 -0.00187100

C -1.64948500 -4.15185100 0.82178400

C -2.88706900 -4.81105400 1.39484200

H -3.77669400 -4.32002900 0.98466300

H -2.93173300 -5.88176300 1.17969700

H -2.90873400 -4.66091500 2.48037800

O 0.85480800 -3.20842600 -0.41788200

O -1.55939000 -2.89756800 1.03948900

Cu -0.12298900 -1.71132200 0.44900100

S -2.75939300 0.29333300 -0.10709200

C -3.07733800 1.39073000 1.29470900

C -4.37640600 1.41324900 1.80620100

C -2.04143300 2.16026600 1.83495600

C -4.65132900 2.25097300 2.88690200

H -5.13923400 0.77954800 1.36736200

C -2.34024600 2.98915300 2.91810100

H -1.03171700 2.09881400 1.43646800

C -3.63584800 3.03684600 3.43919600

H -5.65563100 2.28498900 3.29936000

H -1.55160700 3.59206400 3.35871800

H -3.85476400 3.68445600 4.28389800

C -2.60362000 1.29965100 -1.60268500

C -1.45156900 2.06295200 -1.81757400

C -3.65767800 1.25996100 -2.51765800

C -1.37231300 2.81449300 -2.99146800

H -0.63604100 2.05704400 -1.09887600

C -3.55834000 2.02158300 -3.68209700

H -4.52207500 0.63666600 -2.31674400

C -2.41886400 2.79613800 -3.91701300

H -0.48229500 3.40636600 -3.18382500

H -4.36765400 2.00500600 -4.40644000

H -2.34469800 3.38338600 -4.82832800

O -3.94595700 -0.57702600 -0.28301900

N -1.27436000 -0.14033900 0.16461900

S 1.89817400 0.68277100 0.42032700

C 3.39650800 1.04016900 1.38951500

C 3.67558600 2.35812900 1.75187600

C 4.23216500 -0.01069600 1.77170700

C 4.82171600 2.62815000 2.50300800

H 2.98996700 3.14677800 1.46124700

C 5.37546500 0.27022700 2.52038600

H 3.96792000 -1.02677000 1.49832500

C 5.67191700 1.58728500 2.88306400

H 5.04675800 3.65047000 2.79531900

H 6.03204300 -0.53998100 2.82606700

H 6.56277700 1.80098400 3.46786000

C 2.52587300 0.67176100 -1.30136200

C 2.75062300 -0.55894600 -1.91852000

C 2.76125200 1.87408400 -1.97287700

C 3.22085200 -0.58152500 -3.23438700

H 2.53651300 -1.47576000 -1.37624800

C 3.23947000 1.83999400 -3.28329400

H 2.56041300 2.81653400 -1.47400900

C 3.46969300 0.61307700 -3.91336400

H 3.39252000 -1.53441200 -3.72809600

H 3.42926400 2.76937800 -3.81431800

H 3.83968500 0.59019200 -4.93512800

O 1.08837700 1.95192000 0.48536800

N 1.45511900 -0.70471300 0.83978200

**26-sing**

C -0.04479400 -4.31353200 -1.14769900

C 0.82741900 -5.50911500 -1.47351400

H 1.33557000 -5.84245300 -0.56144300

H 0.25527500 -6.34168400 -1.89024300

H 1.60454600 -5.21071500 -2.18563100

C -1.43593600 -4.40172500 -1.32877800

H -1.84600400 -5.31801500 -1.73529700

C -2.32332600 -3.39599900 -0.92879300

C -3.81669500 -3.58734000 -1.06175100

H -4.23509600 -2.72007000 -1.58272900

H -4.07275200 -4.50697900 -1.59374100

H -4.26880900 -3.61352300 -0.06320700

O 0.58492900 -3.29722500 -0.70336900

O -1.98265400 -2.27676400 -0.41023900

Cu -0.22487300 -1.55639300 -0.28273300

S -2.23984500 0.60876200 -0.38443100

C -2.76479200 0.33420400 1.33821600

C -4.09960300 0.01010700 1.58327000

C -1.83315500 0.44638800 2.37267800

C -4.51164500 -0.20508500 2.89924600

H -4.78707300 -0.08082400 0.74946600

C -2.26102700 0.22969300 3.68448700

H -0.79662300 0.69156100 2.16000700

C -3.59420600 -0.09496400 3.94750700

H -5.54739600 -0.46191700 3.10493800

H -1.54749300 0.31178400 4.49965900

H -3.91868900 -0.26459300 4.97084600

C -2.18463400 2.41589400 -0.54993600

C -1.16119800 3.15717500 0.04450200

C -3.20669700 3.02236000 -1.28070800

C -1.18223100 4.54627800 -0.09075900

H -0.36290900 2.65777600 0.58400800

C -3.20806600 4.41226200 -1.41216200

H -3.97321500 2.40531200 -1.73613500

C -2.20014000 5.17275700 -0.81548400

H -0.39656900 5.14007700 0.36886000

H -3.99641100 4.89840700 -1.98038200

H -2.20605900 6.25490200 -0.91715600

O -3.37845200 0.16333300 -1.23565600

N -0.74233500 0.22035800 -0.60173000

S 1.93849800 0.39464400 0.58126800

C 3.60594600 0.08148800 1.22905000

C 4.13418400 1.00326400 2.13276800

C 4.32217200 -1.05080400 0.83464700

C 5.41668600 0.79086000 2.64263600

H 3.53794900 1.85772800 2.43324800

C 5.60215800 -1.25051000 1.35202000

H 3.86963800 -1.76610700 0.15734200

C 6.14995500 -0.33124700 2.25141900

H 5.83892000 1.50045900 3.34885600

H 6.16914300 -2.12929100 1.05745000

H 7.14704700 -0.49366500 2.65181200

C 2.26138800 1.13759800 -1.05136100

C 2.41603800 0.29953800 -2.15534300

C 2.38964600 2.52181300 -1.16413800

C 2.69990000 0.86383500 -3.39959800

H 2.29605900 -0.77289100 -2.03891500

C 2.67636500 3.07554600 -2.41271900

H 2.25238300 3.14770000 -0.28938100

C 2.83144900 2.24872400 -3.52801800

H 2.81224300 0.22187300 -4.26891700

H 2.77215200 4.15305000 -2.51492100

H 3.05121400 2.68448000 -4.49891300

O 1.38005800 1.49666100 1.42568400

N 1.38430700 -1.04697200 0.47871600

**27-MECP**

C 0.77400500 4.34502400 -1.21908600

C 0.15438100 5.65040200 -1.68113900

H -0.40243800 6.09569600 -0.84798200

H 0.89782000 6.36990800 -2.03387400

H -0.56692700 5.45057200 -2.48062100

C 2.18047900 4.23721200 -1.15955600

H 2.76884100 5.08116700 -1.50048100

C 2.86332100 3.12536500 -0.65250800

C 4.37591000 3.11269700 -0.62111700

H 4.73341500 2.24471500 -1.18640800

H 4.81081400 4.02417100 -1.03794500

H 4.71862100 2.98996700 0.41272700

O -0.05202300 3.43373200 -0.90023000

O 2.31202900 2.07186300 -0.17492100

Cu 0.42776300 1.58239700 -0.31188400

S 2.28893300 -0.96360000 -0.44426600

C 2.71875700 -0.70531200 1.29588100

C 4.07353900 -0.63077500 1.62295700

C 1.71303900 -0.59228300 2.25853700

C 4.43145300 -0.42937000 2.95594700

H 4.82204500 -0.71425600 0.84334100

C 2.09218000 -0.38883900 3.58729100

H 0.66071800 -0.65708200 1.99280300

C 3.44268500 -0.30531200 3.93504900

H 5.48158400 -0.36629600 3.22696900

H 1.32469400 -0.29197300 4.34926100

H 3.72658500 -0.14371200 4.97134500

C 1.85925100 -2.71501800 -0.55523700

C 0.67571000 -3.20680500 -0.00026600

C 2.76503500 -3.53583300 -1.22893900

C 0.41429900 -4.57265200 -0.11712400

H -0.03694400 -2.54889400 0.49103700

C 2.48365300 -4.89884100 -1.33288100

H 3.66160000 -3.10834500 -1.66364000

C 1.31270400 -5.41659800 -0.77540700

H -0.50146400 -4.97430200 0.30669700

H 3.17883400 -5.55308700 -1.85128900

H 1.09785300 -6.47881100 -0.85773000

O 3.51641000 -0.73670500 -1.24724600

N 0.97743600 -0.19122400 -0.92418400

S -2.02238000 -0.07299100 0.51385400

C -3.63300000 0.27661000 1.27738100

C -4.23668500 -0.70435600 2.06502400

C -4.23280800 1.52042400 1.07603200

C -5.47331900 -0.43526400 2.65473600

H -3.72964200 -1.65009700 2.22231600

C -5.46937200 1.77786400 1.66927400

H -3.71868000 2.26973800 0.48332600

C -6.09007900 0.80177600 2.45388100

H -5.94919100 -1.18915800 3.27606400

H -5.94451800 2.74434200 1.52464500

H -7.05194400 1.00876000 2.91550100

C -2.50399900 -0.77552000 -1.10817600

C -2.51610500 0.07269800 -2.21512300

C -2.84384400 -2.12445300 -1.22970600

C -2.86899400 -0.44004900 -3.46491100

H -2.23302300 1.11344200 -2.08929700

C -3.20103600 -2.62789000 -2.48141700

H -2.81367300 -2.76959200 -0.35828300

C -3.21166600 -1.78768400 -3.59807100

H -2.87035800 0.21212400 -4.33401000

H -3.46284400 -3.67762300 -2.58665500

H -3.47997000 -2.18634400 -4.57273700

O -1.48487000 -1.25649300 1.27940600

N -1.32330900 1.26814300 0.38020100

**28-ts**

C 2.96616900 2.63177300 -1.75361200

C 4.30772100 2.50318300 -2.45605100

H 4.19585100 1.85787600 -3.33567000

H 4.71531900 3.46684500 -2.77345500

H 5.02701100 2.01414700 -1.78820000

C 2.38767700 3.90412200 -1.62578900

H 2.93313800 4.74450300 -2.04034900

C 1.14998700 4.17965500 -1.00331000

C 0.66620500 5.61871800 -0.94760000

H 0.48778400 5.90102700 0.09627400

H 1.37260600 6.32335300 -1.39507900

H -0.29526400 5.69418800 -1.46847400

O 2.46241200 1.53696200 -1.32851400

O 0.37395200 3.32741700 -0.46935100

Cu 0.58538800 1.35923200 -0.51818900

S -2.21716200 0.40900000 -0.58991100

C -2.53623500 -1.21949200 -1.35594300

C -2.87523500 -1.23781800 -2.70936800

C -2.47465700 -2.39033000 -0.59969700

C -3.16431800 -2.46157500 -3.31673800

H -2.90799600 -0.30692600 -3.26352600

C -2.77083800 -3.60483100 -1.21872800

H -2.16338100 -2.36567100 0.43753700

C -3.11639600 -3.64166900 -2.57243100

H -3.42802400 -2.48910600 -4.37038300

H -2.72088500 -4.52432600 -0.64230400

H -3.34431900 -4.59211700 -3.04762200

C -3.54711700 0.62453400 0.60937900

C -3.47475400 0.04660900 1.88025700

C -4.64032500 1.39467300 0.21093000

C -4.53666300 0.24126400 2.76292100

H -2.59766400 -0.52099600 2.16948900

C -5.69469000 1.57968000 1.10687100

H -4.64634700 1.84572300 -0.77547900

C -5.64426400 1.00269200 2.37748000

H -4.49553100 -0.19719800 3.75601700

H -6.55141400 2.17915100 0.81241100

H -6.46658400 1.15080300 3.07214200

O -2.41639800 1.38308700 -1.69472800

N -0.96498700 0.46528800 0.37297200

S 0.85584500 -1.45671400 0.76142600

C 2.15139100 -2.46433500 0.00970500

C 2.22260500 -3.80178700 0.40243900

C 3.03727600 -1.91585100 -0.92353500

C 3.21535600 -4.61273500 -0.14919100

H 1.50354000 -4.19065000 1.11512700

C 4.02274900 -2.74359800 -1.46368400

H 2.94797900 -0.87745200 -1.22930000

C 4.11405900 -4.08388600 -1.07826900

H 3.28232100 -5.65657200 0.14422100

H 4.71758200 -2.33831800 -2.19384400

H 4.88510200 -4.71860500 -1.50663300

C 1.67950100 -0.53399700 2.09918800

C 2.64766400 0.42959800 1.80278900

C 1.31497000 -0.82436300 3.41459200

C 3.25436400 1.11618800 2.85571300

H 2.91697300 0.65847000 0.77615500

C 1.92850600 -0.12620900 4.45629700

H 0.56151300 -1.58001800 3.60440100

C 2.89589100 0.84133700 4.17791500

H 4.00451700 1.87109400 2.63819200

H 1.64796400 -0.33882800 5.48414100

H 3.37064500 1.38301100 4.99148100

O -0.08783900 -2.37378400 1.46212400

N 0.43295800 -0.57192400 -0.47232000

**29**

C -2.98903000 3.32354200 -1.31583100

C -3.39502300 4.78783900 -1.26325500

H -2.50990200 5.41440700 -1.42468500

H -4.15492300 5.04657200 -2.00577400

H -3.77555300 5.02416000 -0.26267800

C -3.57639200 2.50175200 -2.29635300

H -4.28435300 2.97890700 -2.96546900

C -3.32277600 1.12904200 -2.51296000

C -4.03813400 0.44218100 -3.67055000

H -4.56081800 -0.44848800 -3.30205800

H -4.75543200 1.09229900 -4.17956400

H -3.29460500 0.09928100 -4.40064300

O -2.12677600 2.98563900 -0.43738900

O -2.54010900 0.40053800 -1.83020100

Cu -1.26837800 1.28288400 -0.27020600

S 2.14740200 0.81341400 -0.45338500

C 2.66031700 1.31098500 1.22172500

C 2.86450300 2.66748200 1.48219000

C 2.83818900 0.33854000 2.20767000

C 3.27503900 3.05389600 2.75899100

H 2.69113300 3.39453400 0.69674100

C 3.24910500 0.73976800 3.47937600

H 2.62585800 -0.70406600 1.99913500

C 3.47116400 2.09206000 3.75293000

H 3.43696900 4.10586800 2.97663300

H 3.38492600 -0.00498300 4.25844900

H 3.79004800 2.39763500 4.74588800

C 3.58761100 0.06563700 -1.22589700

C 3.92233600 -1.27282600 -1.00678800

C 4.36116200 0.89362700 -2.03999600

C 5.06565500 -1.78476300 -1.62001600

H 3.28489600 -1.89675900 -0.39169200

C 5.50449900 0.36770700 -2.64361400

H 4.05374700 1.92066000 -2.20528400

C 5.85666500 -0.96677500 -2.43219400

H 5.33571400 -2.82571300 -1.46669600

H 6.11397700 0.99940900 -3.28340400

H 6.74645700 -1.37313200 -2.90530900

O 1.84669500 2.08709800 -1.15584500

N 1.14952400 -0.41253400 -0.47036600

S -0.41724300 -1.47183700 1.09666700

C -1.98811400 -1.09420900 1.88126200

C -1.99479400 -1.15683100 3.27580400

C -3.13465800 -0.77735900 1.14552100

C -3.18001300 -0.87309700 3.95523500

H -1.08546600 -1.41860300 3.80527800

C -4.31024300 -0.49828400 1.84463900

H -3.11877300 -0.72885200 0.06176500

C -4.33351200 -0.54302500 3.24090200

H -3.19905500 -0.91028400 5.04051100

H -5.20769400 -0.24014700 1.29043800

H -5.25470700 -0.32240200 3.77312700

C -0.71247300 -2.82624900 -0.07191900

C -1.27114100 -2.55456000 -1.32376100

C -0.36971500 -4.12121000 0.32451300

C -1.49645600 -3.62612500 -2.19043600

H -1.52879600 -1.54169000 -1.62333400

C -0.60293700 -5.17732100 -0.55648600

H 0.07944600 -4.28470400 1.29766000

C -1.16614400 -4.92928300 -1.81095700

H -1.92681700 -3.43500400 -3.16927500

H -0.34123000 -6.19040100 -0.26409700

H -1.34451200 -5.75413300 -2.49566500

O 0.51610900 -2.01612000 2.11802800

N -0.05175500 -0.12936900 0.30583400

**30-MECP**

S -0.76389300 1.90831000 -0.97706600

C -2.53240600 2.03074000 -0.56687500

C -3.43347000 2.14307600 -1.62591500

C -2.96961600 1.95261000 0.76009400

C -4.80241300 2.16649300 -1.34824500

H -3.05684400 2.20618100 -2.64138700

C -4.33867500 1.98571800 1.02286900

H -2.25573900 1.87018400 1.57379700

C -5.25422000 2.08729000 -0.02970000

H -5.51453500 2.24626700 -2.16471900

H -4.68984400 1.92895000 2.04932900

H -6.32011400 2.10758600 0.18114500

C -0.02975100 3.25971400 -0.02252600

C 0.50838600 3.02396300 1.24330700

C -0.07596100 4.53991600 -0.57767000

C 0.98940200 4.11043800 1.97646400

H 0.58992800 2.01226200 1.62579600

C 0.40982600 5.61506500 0.16747900

H -0.47295200 4.68341900 -1.57737000

C 0.93417600 5.40131600 1.44475000

H 1.41908000 3.94216600 2.96007600

H 0.38288100 6.61726900 -0.25160700

H 1.30737100 6.24144700 2.02426900

O -0.63970100 2.24329500 -2.41414300

N -0.28439900 0.52953100 -0.41472000

S 2.32792700 -0.19400800 0.27283200

C 1.85112500 -1.90579600 -0.14937100

C 1.88373300 -2.89606300 0.83540300

C 1.49632300 -2.20071600 -1.46735300

C 1.56982100 -4.21039800 0.48454100

H 2.13825900 -2.62930600 1.85554000

C 1.18055400 -3.51848900 -1.80562700

H 1.44553000 -1.39996800 -2.19845000

C 1.22339200 -4.52136000 -0.83375300

H 1.59259200 -4.99077200 1.24043500

H 0.89558000 -3.75870500 -2.82611300

H 0.98061300 -5.54642500 -1.10181800

C 4.12077300 -0.18788800 -0.00931200

C 4.64312900 -0.25667300 -1.30363100

C 4.94919800 -0.07870200 1.10717300

C 6.02661200 -0.23229200 -1.47380600

H 3.97704100 -0.31390000 -2.15735800

C 6.33308800 -0.04903800 0.92215000

H 4.50221900 -0.01482300 2.09336800

C 6.87076800 -0.13009200 -0.36391400

H 6.44655700 -0.28774500 -2.47430100

H 6.98978600 0.03744400 1.78336400

H 7.94840800 -0.10792200 -0.50305600

O 2.11682600 -0.09204000 1.74837600

N 1.82896900 0.84031900 -0.77465600

C -3.18242100 -2.93196400 -0.64309600

C -3.93524900 -3.66651900 -1.73572200

H -3.21508500 -4.14952900 -2.40671100

H -4.61718900 -4.42361900 -1.34036300

H -4.50146700 -2.94737000 -2.33805000

C -3.36959800 -3.32007500 0.69719700

H -4.06411200 -4.12955800 0.89036200

C -2.71802000 -2.74863400 1.80314700

C -3.01539500 -3.27457800 3.19298700

H -3.41368300 -2.46359600 3.81423300

H -3.72825900 -4.10307600 3.18588000

H -2.08065700 -3.60445200 3.66026600

O -2.41259300 -1.99992700 -1.05193900

O -1.86436600 -1.79762500 1.76006600

Cu -1.31454800 -0.89628400 0.14109200
